# Enhancing tumor-specific recognition of programmable synthetic bacterial consortium for precision therapy of colorectal cancer

**DOI:** 10.1038/s41522-024-00479-8

**Published:** 2024-01-20

**Authors:** Tuoyu Zhou, Jingyuan Wu, Haibo Tang, Dali Liu, Byong-Hun Jeon, Weilin Jin, Yiqing Wang, Yuanzhang Zheng, Aman Khan, Huawen Han, Xiangkai Li

**Affiliations:** 1 Ministry of Education Key Laboratory of Cell Activities and Stress Adaptations, School of Life Sciences, Lanzhou University, Lanzhou, China; 2 The First Clinical Medical College of Lanzhou University, Lanzhou University, Lanzhou, China; 3 Department of Chemistry and Biochemistry, Loyola University Chicago, Chicago, IL USA; 4 Department of Earth Resources and Environmental Engineering, Hanyang University, Seoul, Korea; 5 Medical Frontier Innovation Research Center, The First Hospital of Lanzhou University, Lanzhou, China; 6 Discovery Biology, Curia Golbal Inc, New York, NY USA; 7 State Key Laboratory of Grassland Agro-ecosystems, College of Pastoral Agricultural Science and Technology, Lanzhou University, Lanzhou, China

**Keywords:** Applied microbiology, Microbiome

## Abstract

Probiotics hold promise as a potential therapy for colorectal cancer (CRC), but encounter obstacles related to tumor specificity, drug penetration, and dosage adjustability. In this study, genetic circuits based on the *E. coli* Nissle 1917 (EcN) chassis were developed to sense indicators of tumor microenvironment and control the expression of therapeutic payloads. Integration of XOR gate amplify gene switch into EcN biosensors resulted in a 1.8-2.3-fold increase in signal output, as confirmed by mathematical model fitting. Co-culturing programmable EcNs with CRC cells demonstrated a significant reduction in cellular viability ranging from 30% to 50%. This approach was further validated in a mouse subcutaneous tumor model, revealing 47%-52% inhibition of tumor growth upon administration of therapeutic strains. Additionally, in a mouse tumorigenesis model induced by AOM and DSS, the use of synthetic bacterial consortium (SynCon) equipped with multiple sensing modules led to approximately 1.2-fold increased colon length and 2.4-fold decreased polyp count. Gut microbiota analysis suggested that SynCon maintained the abundance of butyrate-producing bacteria *Lactobacillaceae* NK4A136, whereas reducing the level of gut inflammation-related bacteria *Bacteroides*. Taken together, engineered EcNs confer the advantage of specific recognition of CRC, while SynCon serves to augment the synergistic effect of this approach.

## Introduction

Malignant neoplasms pose a huge challenge on global health, affecting the health and life quality of individuals worldwide^1^. Colorectal cancer (CRC) stands out as one of the most prevalent life-threatening cancers, with genetic and environmental risk factors contributing to its onset^2^. The survival rate for advanced stage colorectal cancer remains low^3^ despite the availability of treatment options, such as minimally invasive surgery, chemotherapy, radiation therapy, and immunotherapy^4^. The drawbacks of these regimens mainly include nonspecific toxicity to rapidly dividing normal cells^5^, multidrug-resistant cell formation^6^ and cytokine storm^7^. Even targeted therapies (e.g. Bevacizumab or Aflibercept) with low side effects often come with limited practical treatment options^8^. Furthermore, the occurrence of metastasis and its recurrence severely limits conventional treatment options for CRC^9^. Hence, there is a dire need to develop novel therapies to complement or substitute traditional therapies for CRC treatment.

Due to inhibition of intratumor immune surveillance and availability of necrotic tumor core nutrients, the ability of bacteria to selectively homing tumors promotes novel models of cancer treatment and diagnosis^10,11^. Natural bacteria, such as Coley toxin, have been used to treat patients with malignant tumors since 100 years ago^12^. The *Bacillus* Calmette-Guérin, the anti-tuberculosis vaccine, is also used to treat non-muscular invasive bladder cancer^13^. Some anaerobic bacteria, such as *Salmonella*^14,15^ and *Clostridium*^16,17^, can selectively invade oxygen-deprived areas of tumors and destroy cancer cells. However, the risk of toxicity and infection has hindered the clinical application of natural bacteria as anticancer agents^18,19^.

Probiotics have been used to ameliorate human diseases, with successful applications in mitigating hyperuricemia^20,21^ and alleviating heavy metal toxicity^22^. Nevertheless, the inadequacy of innate therapeutic potency of natural probiotic remains an obstacle to their applications on CRC treatments^23,24^. Synthetic gene circuits have been designed to improve the application potential of bacteriotherapy^25–27^. In an impressive study, utilizing a synchronous bacterial lysis cycle for *S. typhimurium* modification continuously delivered therapeutic drugs in tumor regions^28^. Engineered *E. coli* harboring myrosinase and tumor-targeting adhesion protein HlpA could prevent carcinogenesis and promote CRC regression through cruciferous vegetable diet^29^. However, the vast majority of engineered bacteria are adopted constitutive expression systems to deliver payloads; Some payloads are toxic to normal cells^30–33^, which increases the exposure risk to non-targeted organs^23,34^.

*E. coli* Nissle 1917 (EcN) are generally considered safe and beneficial to host health^35^. Furthermore, oxygen^36^, pH^37^, and lactate^38^ can be used as indicators of tumor microenvironment (TME) uniqueness. By employing genetic circuit programming to perceive these physiological characteristics, it may enhance EcN’s specific recognition of tumors^39^ and strictly control the production of payloads^40^. On the other hand, single cellular chassis often faces limitations such as foreign DNA burden, metabolic crosstalk, and intra-cellular resource competition^41^. Synthetic bacterial consortium (SynCon) can alleviate the metabolic load on individual chassis^42^ and achieve division of labor among multiple strains^43^. We further hypothesized that SynCon could enhance the therapeutic efficacy of CRC through synergies. To verify these concepts, the performance of biosensors was accessed in simulators and cell culture mediums. Subsequently, in vitro co-culture assays were conducted to evaluate the cytotoxicity of modified EcNs towards tumor cells. Further, the therapeutic effects of the engineered EcNs and SynCons were validated using the CT26 homograft mouse model and azoxymethane (AOM)/dextran sulfate sodium (DSS) induced colitis-associated mouse tumorigenesis, respectively. Considering the notable interaction between gut microbiota and individual’s response to medications, the distribution of microbiota during the administration of SynCons in AOM/DSS model were also examined to elucidate the intricate interactions among specific microbial constituents.

## Result

### Engineered biosensors can recognize the tumor microenvironment

**Fig. 1:**
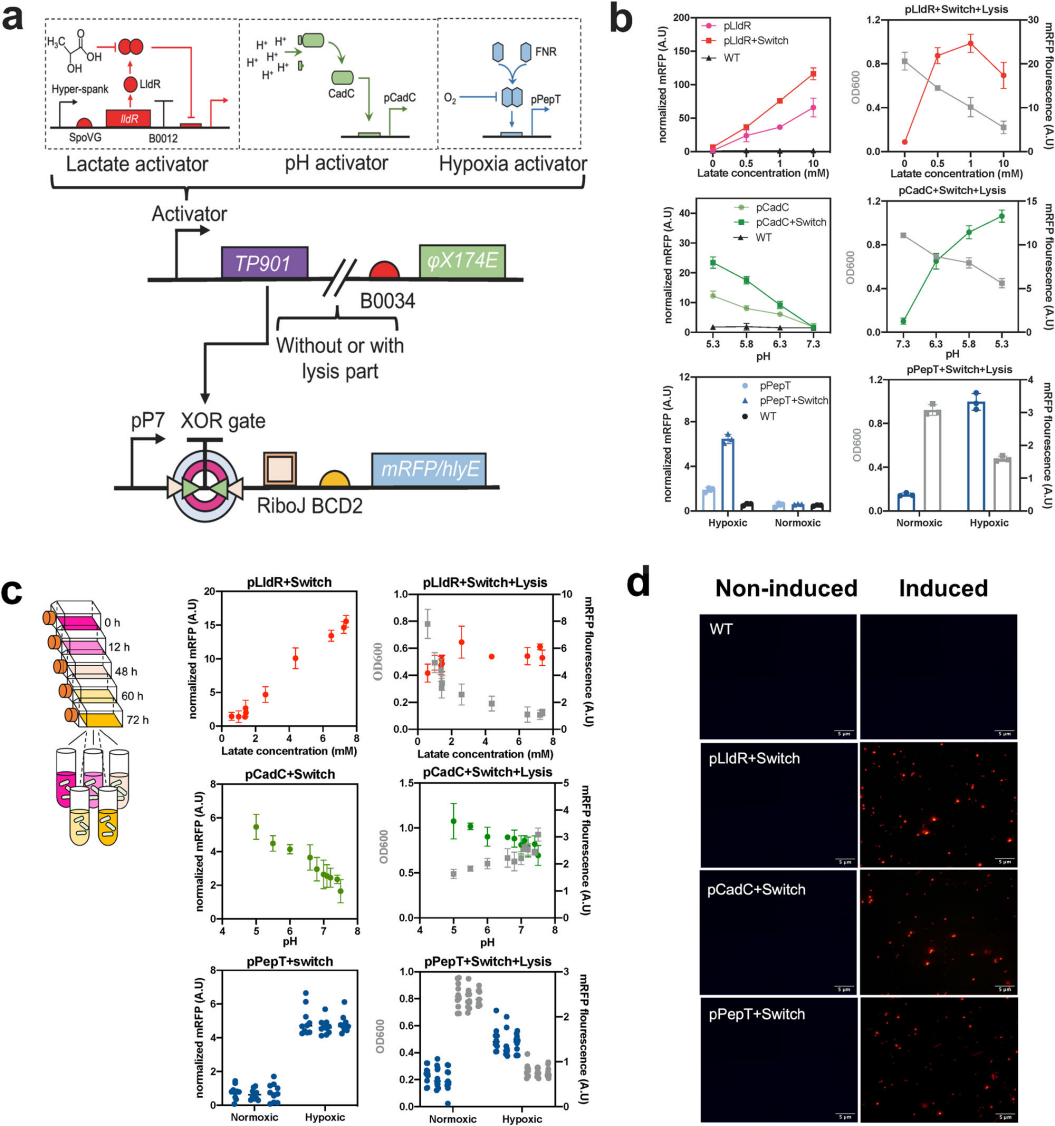
Design of gene circuit and characterization of biosensor strains. **a** Construction diagram of engineered bacteria. Programmable bacterial circuits can sense specific environmental signals and increase the transmission function of biosensors by amplifying gene switch. When lactate, H^+^ are present, or in anoxic condition, serine integrase TP901 driven by inductive promoter pLldR, pCadC, and pPepT can reverse the terminator orientation of the XOR gate, enabling the strong promoter pP7 transcript downstream genes. When *φ*X174E is added to the circuit, engineered EcN would lyse itself with above environmental factors induction. **b** Relationship between environmental change and biosensor fluorescence or population intensity. Biosensor strains harboring mRFP were grown in specified environmental condition (0–10 mM lactate, pH 5.3, 5.8, 6.3 and 7.3, and 0% or 20% oxygen) for 48 h and their induction fluorescence signal were assayed (*n* = 3 biological replicates. Data are shown as mean ± s.e.m). **c** Biosensor strains responded to physiological cues. Cell culture medium supernatant from CT26 cancer cell line was collected every 12 h over 5 d and then cultured with biosensor strains. The hypoxic biosensor co-cultured with cell medium supernatant was grown under conditions with or without oxygen. After incubation at 37 °C for 12 h, mRFP fluorescence intensity of lactase (red), pH (green) and hypoxic (blue) biosensor strains was measured and recorded (*n* = 3 biological replicates. Data are shown as mean ± s.e.m). **d** Fluorescence microscopy observation. The fluorescence signals of lactate, pH and hypoxic biosensors under induced (10 mM, pH 5.3 and 0% O_2_, respectively) and noninduced (0 mM, pH 7.3 and 20% O_2_, respectively) conditions were observed using Olympus Bx53 (magnification: 40x). Bacteria were cultured in LB medium at 37 °C for 12 h. The scale bar marked in the lower right corner of the image represents 5 μm.

A dual-plasmid architecture was devised to sense the TME and control the expression of therapeutic payloads (Fig. 1a and Supplementary Fig. 1). The genetic circuit consisted of two main parts: the sensing module and the working module. The sensing module I was comprised of L-lactate/H^+^/hypoxic inducible promoters (pLldR, pCadC and pPepT) and the serine integrase coding gene *TP901*, whereas the sensing module II was additionally coupled with the lysis gene *φX174E* downstream of *TP901*. The working module introduced coding genes of mRFP or therapeutic payloads downstream of XOR gate. The XOR gate consisted of the recognition site of serine integrase and a terminator sequence, and formed an amplifying gene switch (XOR Switch) with *TP901*. When environmental factors are present, the induction module produces TP901, which recognizes and flips the termination sequences of the XOR gate, allowing expression of downstream products. In addition, sensing module II encoded *φ*X174E and triggered bacterial lysis. As for ten mutants of the lactate-responsive operon pLldR (Supplementary Fig. 2), the mutant pLldR10 was selected for further study owing to its highest fold change and the lowest noise. Besides, pCadC^44^ and pPepT^45^ were chosen based on previous studies. As expected^39^, mRFP reporter system suggested pLldR and pCadC is regulated by lactate and H^+^, respectively, while pPepT synthesized more fluorescent proteins under anoxic conditions (Fig. 1b left). Among them, the pLldR maintained the maximum transcriptional strength. Subsequently, the effect of XOR Switch on promoter transcription levels were tested. At 10 mM lactate, pLldR transcribed mRFP to produce a normalized fluorescence density of 70 A.U., which is increased to 120 A.U. with the addition of XOR Switch (Fig. 1b left). Similarly, the XOR Switch enhanced the transcription levels of pCadC and pPepT by approximately 1.9-fold and 2.8-fold, respectively. The characteristics of the lysis biosensors (sensing module II) were also tested. Under the same culture conditions, the bacterial density of biosensors supplemented with *φ*X174E gradually decreased with increasing induction intensity (Fig. 1b, right). The fluorescence intensity of pLldR induced lysis biosensor peaked at 1 mM lactate (25 A.U), and then decreased to 18 A.U at 10 mM lactate. The tracking analysis of bacterial population dynamics observed the periodic variation of lysis biosensor (Supplementary Fig. 3). As a pivotal stride towards in vivo characterization of biosensors, the capacity of bacterial biosensors to perceive cellular metabolic activity was assessed within the supernatant of cell cultures (Fig. 1c, left). The fluorescence levels of pLldR and pCadC-controlled biosensors were increased with lactate and H^+^ concentration, while the fluorescence signal of pPepT based biosensor showed an increasing trend with decreasing oxygen content (Fig. 1c, middle). Similarly, three lysis biosensors shared the same responses trend with induction signals, but exhibited the lower fluorescence intensity than biosensors without the lysis gene (Fig. 1c, right). Furthermore, the OD_600_ of lysis biosensors exhibited a concomitant decline as the corresponding induction signal in the cell culture supernatants intensified. Fluorescence imaging further confirmed the six engineered biosensors can detect and react to specific biochemical signals in the host environment (Fig. 1d and Supplementary Fig. 4).

### Biosensor behavior can be predicted using mathematical models

**Fig. 2:**
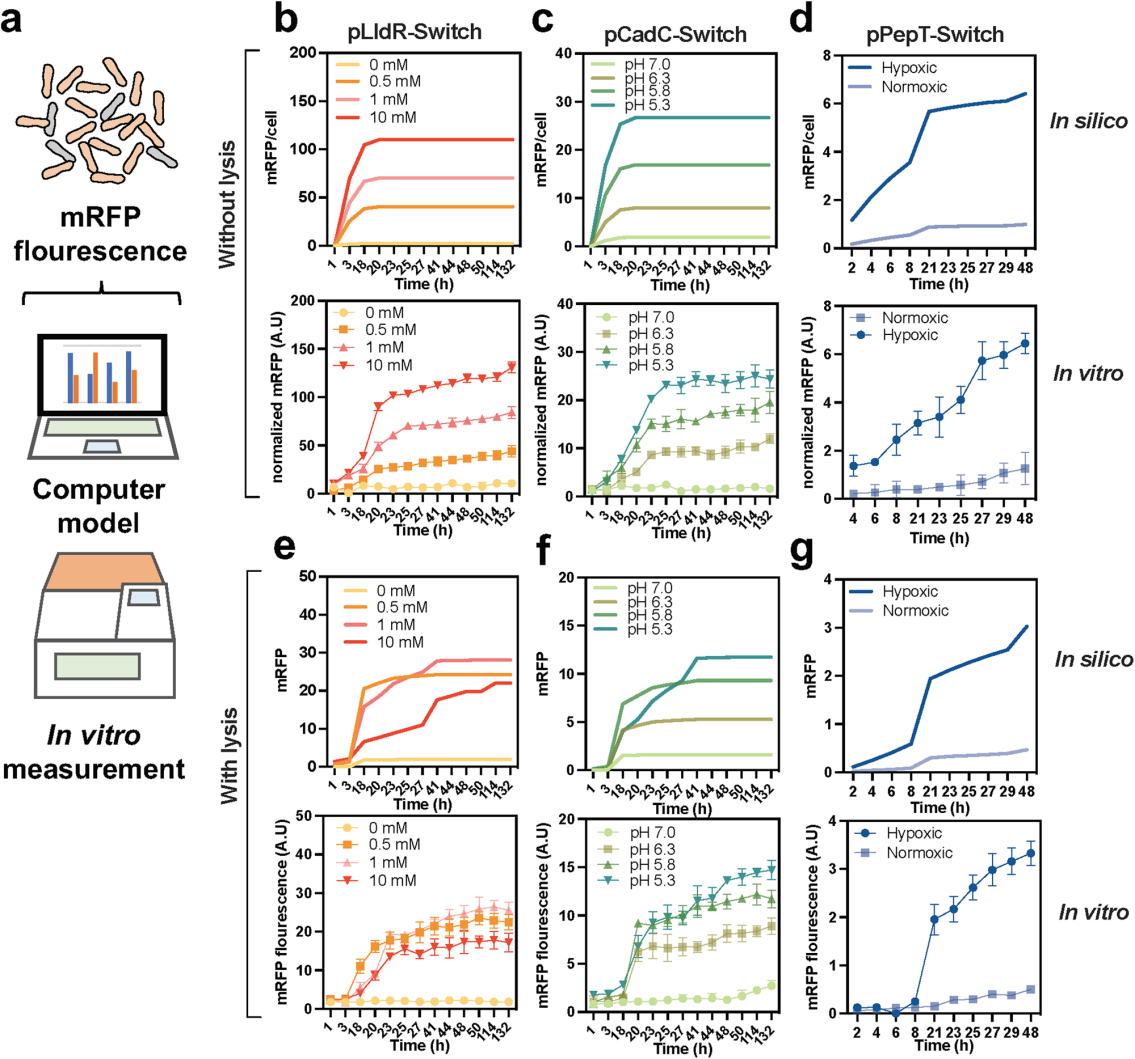
Computational modelling of biosensor strains. **a** Fluorescent changes of biosensor strains under varying environmental signals were measured using microplate reader or predicted in silico modelling. **b**–**d** Biosensor strains were modelled (up) under regulation of pLldR (lactate response), pCadC (pH response) and pPepT (hypoxic response) promoters and compared with (down) in vitro experimental results (*n* = 3, ± s.e.m). The fluorescent results of mRFP were normalized by OD_600_. **e–g** In presence of lysis gene, in vitro mRFP fluorescence of biosensor strains were compared with mathematic modeling prediction (*n* = 3, ± s.e.m). Detailed equations and parameters used in this study could be obtained in supplementary materials.

To bridge the temporal gap between bacterial cloning and animal experimentation, it is necessary to engage in predictive modeling of bacterial therapy prior to in vivo testing (Fig. 2a). Based on the Hill equation^28,46^, which describes promoter activity through repressor or activator occupancy, a system of ordinary differential equations was built upon prior work^39^. The equations detail the response of six biosensors to ambient lactate, oxygen, and H^+^ via XOR Switch. In actual measurements, the peak of biosensors fluorescence was observed at approximately 20 hours after induction, and a detectable signal persisted for at least 48 hours (Fig. 2b-g down). The reaction time for the lactate and pH biosensors could be extended up to 132 hours. These observations were consistent with the results predicted by the computer model (Fig. 2b-g up). After the parameter variables of *φ*X174E were introduced, the total amount of fluorescent protein produced by the model was no longer linearly related to the inducer concentration (Fig. 2e-g up). In the course of time, the results of model fitting present fluctuations. This variability can be ascribed to the influence of lysis genes on the bacterial population (Supplementary Fig. 5)^28,47^. The time progression curve predicted by the simplified model aligns only approximately with actual measurements, possibly due to external environmental disturbances (Fig. 2e-g down). Nevertheless, the predictability of biosensors in terms of their community size and expression level is necessary for the successful implementation of the platform in vivo*’*s complex and fluctuating conditions.

### Therapeutic strains exhibit inhibitory effects on tumor cell activity in vitro

**Fig. 3:**
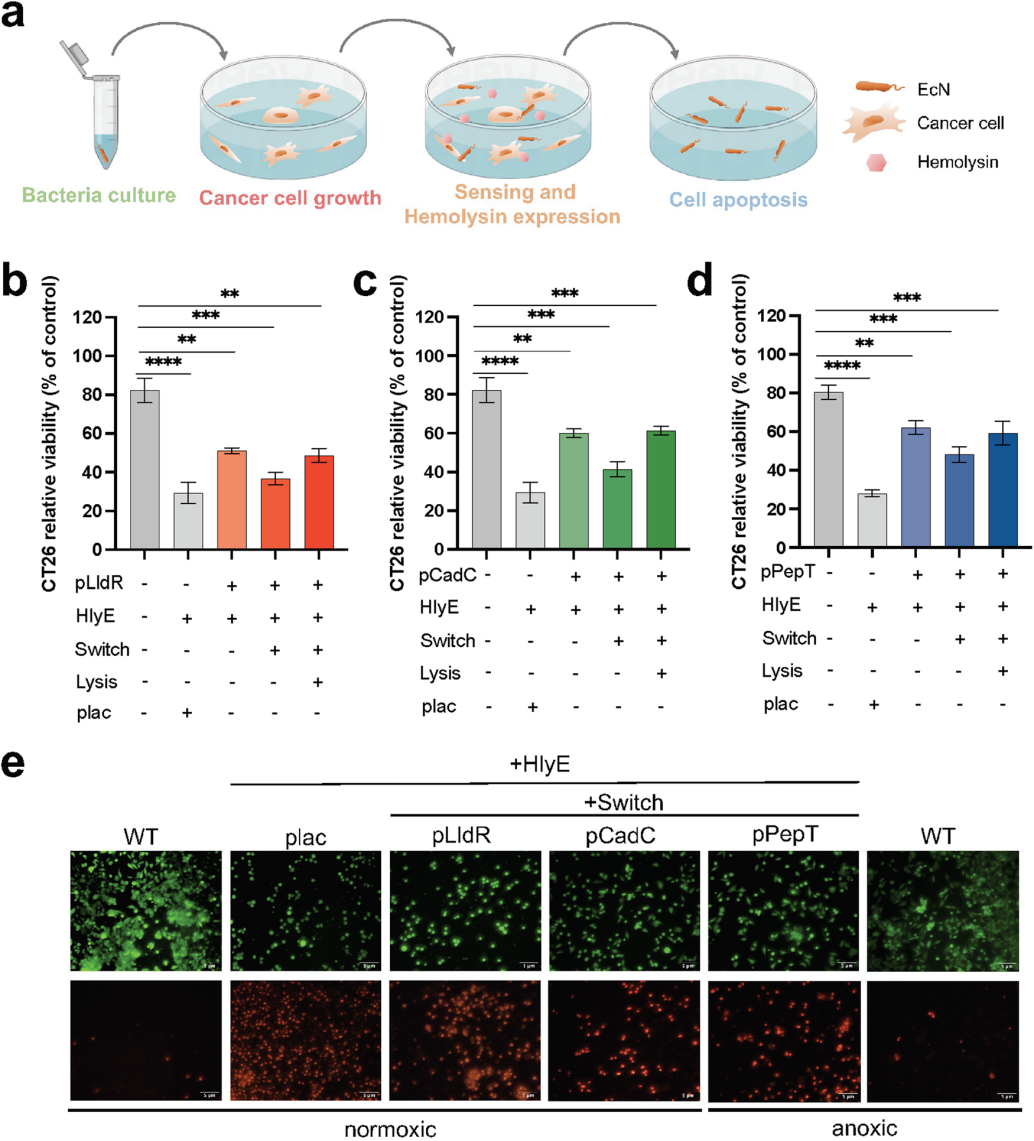
In vitro co-culture of therapeutic strains with cancer cells. **a** Diagram of the co-culture of therapeutic strains and cancer cells. Cancer cells were inoculated in 96-well plates for adherence, then bacteria cultures were seeded for diffusion of released therapeutic. **b**–**d** CT26 cell viability after 3 h co-culture with 30 μL therapeutic strains controlled by pLldR, pCadC and pPepT promoters. “plac” is a constitutive expression promoter, and HlyE represents hemolysin. “Switch” refers to the XOR gate amplifying gene switch, while “lysis” represents the cell lysis element encoded by *φX174E* (*n* = 3, ± s.e.m; One-way ANOVA with Tukey post-test; ** *p* < 0.01, *** *p* < 0.001, and **** *p* < 0.0001). **e** After co-culturing with 30 μL of therapeutic strains for 3 hours, the CT26 cell line was stained with Calcein/PI. The live cells (green fluorescence, stained by Calcein) and dead cells (red fluorescence, stained by PI) were observed using a fluorescence microscope (40x magnification). The oxygen concentration under normoxic conditions is 20%, while under anoxic conditions, it is 0%. The scale bar marked in the lower right corner of the image represents 5 μm.

Next, colorectal cancer cells were co-incubated with therapeutic strains expressing hemolysin (Fig. 3a). A loss of 50%-65% activity was observed in CT26 cells exposed to therapeutic strains harboring *hlyE* (encoding hemolysin) and environmental response promoters, under induction conditions (Fig. 3b-d and Supplementary Fig. 6). The stronger cytotoxicity mediated by pLldR than that by pCadC and pPepT is consistent with fluorescence measurements (Fig. 1) and mathematical model fitting **(**Fig. 2). Furthermore, the XOR Switch enabled therapeutic strains to produce more intense cytotoxicity (25-45%). Despite being attenuated, the incorporation of the lysis gene retains the therapeutic strains’ ability to inhibit CT26 cell activity. In contrast to the inducible system, the activity of CT26 cells only remained 25% when exposed to constitutive therapeutic EcN under the control of plac promoter^48^, while remained 75% under the treatment of equivalent doses of wild-type EcN. Comparable results were obtained by co-culture with RKO and SW480 cells, indicating the broad applicability of this approach (Supplementary Fig. 6). Besides, therapeutic strains exhibited time-dependent and dose-dependent cell mortality on RKO and SW480 cell (Supplementary Fig. 7). Live and dead cell staining also revealed therapeutic EcNs accelerated the death of CT26 cells (Fig. 3e and Supplementary Fig. 8).

### Therapeutic strains suppress the growth of subcutaneous tumors in mice

**Fig. 4:**
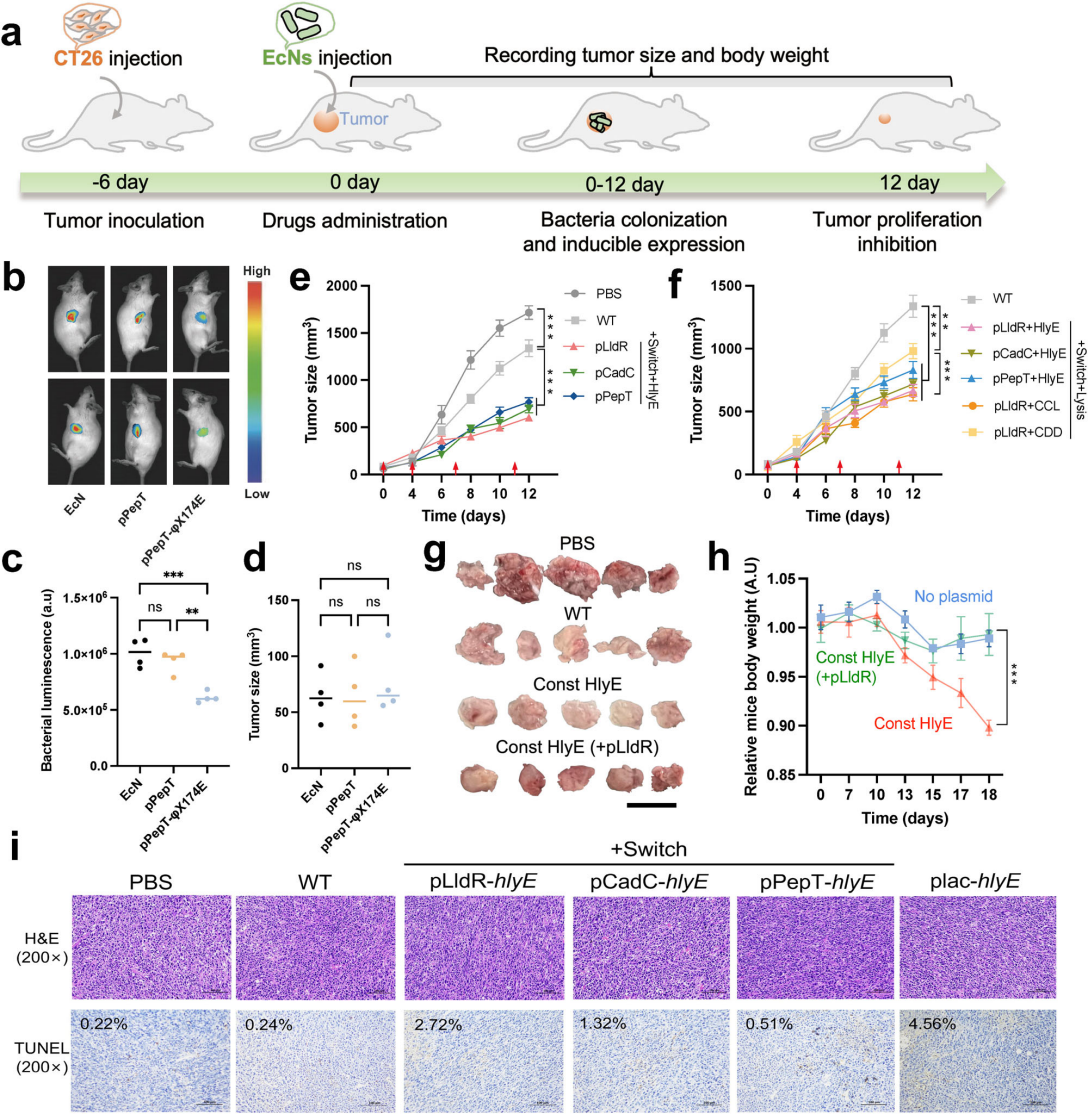
In vivo therapeutic effect on subcutaneous tumor model. **a** Therapeutic schedule of engineered strains administration in a CT26 mouse subcutaneous tumor model. **b** In vivo imaging of mice with dual hind flank tumors injected with luciferase reporter strains using chemiluminescence imager (Vilber, fx6). **c** Integrated luminescence density of the *lux*CDABE reporter strains in a single tumor after injection for 2d was analyzed and calculated using software imageJ 1.53. **d** Dimension of subcutaneous tumor in mice after injection of luciferase reporter strains (*n* = 4, ± s.e.m; One-way ANOVA with Tukey post-test; ns: no significant, **p < 0.01, ***p < 0.001). **e**, **f** Average tumor volume over time for subcutaneous tumor bearing mice injected with wild-type or engineered EcNs. When average tumor volume reached 150 mm³, the engineered bacterial strain was injected intratumorally on day 0 (start of bacterial therapy), 4, 7 and 11, indicated by red arrows (*n* = 10 tumors, error bars represent s.e.m; One-way ANOVA with Tukey post-test; **p < 0.01, ***p < 0.001). CCL is an abbreviation for CCL21 and CDD is an abbreviation of fusion protein CDD_iRGD. **g** Photograph of harvested tumor tissues after different treatments. Scale bar: 2 cm. **h** Average body weight relative to PBS group for mice bearing subcutaneous tumors injected with the therapeutic strain expressing HlyE under control of pLldR (emerald), strain with constitutively expressing HlyE (orange), or the no-plasmid control strain (sapphire). From the date of tumor cell injection, the engineered strains were injected on day 6, 10, 11 and 15 (*n* = 5 mice, error bars show s.e.m). **i** H&E staining and TUNEL staining of the same tumor sections; Scale bars (100 μm) labeled at bottom right of images; TUNEL staining positive rate shown in upper left of images (black). In the TUNEL stained sections, brown represented positive cells. The ratio of TUNEL-positive cells to total cells was analyzed using Aipathwell software. The red box marks the magnified region, highlighting the representative area of positive cells, and is displayed below.

Using a subcutaneous model of CRC in Balb/c mice, the therapeutic potential of programable EcNs were evaluated via intratumoral injection (Fig. 4a). To track bacterial population in mouse homograft tumors, *lux*CDABE was utilized as in vivo reporter gene^49^ (Fig. 4b). Consistent with in vitro measurements (Fig. 1), the luminescence intensity of reporter strain with lysis gene was about 50% compared to that only harboring inducible promoter, demonstrating a notable reduction in bacterial population within tumors (Fig. 4c, d). Compared to the PBS group, the wild-type EcN (WT) group exhibited a 22% reduction in tumor size (*p* = 0.001, One-way ANOVA with Tukey post-test). Relative to the WT group, therapeutic strains harboring inducible promoters and *hlyE* significantly reduced subcutaneous tumor size (Fig. 4e, Supplementary Figs. 9 and 10). Specifically, the pLldR-controlled strain achieved a 54.77% reduction (*p* < 0.001, One-way ANOVA with Tukey post-test), while the pCadC and pPepT strains reduced tumor sizes by 47.95% (*p* < 0.001, One-way ANOVA with Tukey post-test) and 42.51% (*p* < 0.001, One-way ANOVA with Tukey post-test), respectively. Western blot analysis confirmed that HlyE can be detected in tumor tissues (Supplementary Fig. 11). The tumor’s response to bacteria modified with the lysis gene was also notably superior to that observed with unmodified bacteria (Fig. 4f). In the WT group, the average volume of subcutaneous tumors was 1337 mm³. In contrast, tumors treated with lysing strains had an average volume ranging between 638 and 830 mm³. Since the lysis module enables engineered bacteria to release various payloads in tumor grafts^50^, as a prototype validation, two additional therapeutic strains based on pLldR-regulated lysis strains were constructed to activate host immune response (mouse CCL21)^51^ or initiate programmed cell death in tumor cells (CDD-iRGD)^52^. Relative to the WT group, hemolytic or immune-recruiting strains reduced tumor growth by 61.02% and 52.26% (*p* < 0.001, One-way ANOVA with Tukey post-test), while apoptotic strains achieved only a 26.6% reduction (*p* < 0.001, One-way ANOVA with Tukey post-test, Fig. 4f).

EcN that constitutively expresses hemolysin (plac-*hlyE*) showed a stronger tumor suppressive effect than those with promoter-controlled *hlyE* expression (Fig. 4g and Supplementary Fig. 9). However, intratumoral administration of plac-*hlyE* significantly reduced mouse weight to 90% of its day 0 value by the experiment’s end, underscoring the potential systemic impacts of bacterial therapy (Fig. 4h). In contrast, treatment with inducible strains shared comparable body weight changes with unmodified bacteria (Supplementary Fig. 12), suggesting their safety for therapeutic use. Although additional investigations are necessary to comprehensively investigate the impact of these bacteria on host health, these preliminary experiments indicate that using inducible promoters to regulate the payload may alleviate the burden of bacterial injections. Post-mortem histopathological analysis was conducted on residual tumors of mice. TUNEL staining showed increased apoptotic activity in tumor treated with therapeutic strains (Fig. 4i and Supplementary Fig. 13). In addition to the tumor tissue, these therapeutic EcNs were also distributed in the liver and spleen (Supplementary Fig. 14).

### Oral administration of synthetic bacterial consortiums ameliorates the AOM/DSS-induced mouse colorectal cancer

**Fig. 5:**
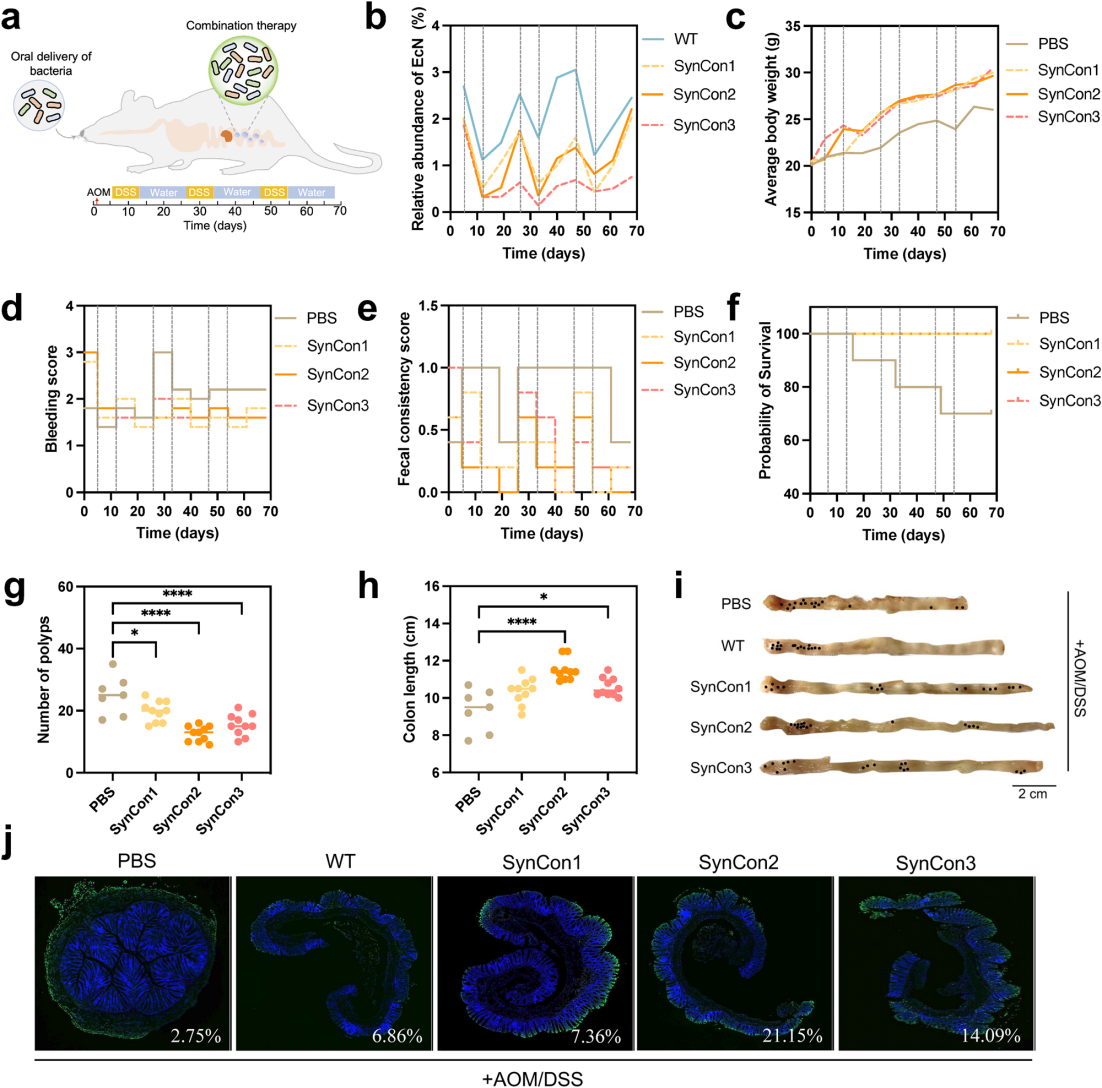
Treatment effects of synthetic bacterial consortium on AOM/DSS-induced colitis-associated-CRC murine model. **a** Schematic diagram of animal treatment scheme. Colon carcinogenesis of mice (*n* = 10) was induced by intraperitoneal injection of azoxymethane (AOM), followed administration of 2% w/v dextran sodium sulfate (DSS) and regular water (repeated three times over 68 days). Mice were orally given therapeutic strain combinations (1 × 10^9 ^c.f.u for each mouse) 6 times a week. **b** Temporal dynamics of the EcN population during the whole experimental period (Black dashed lines represent the DSS treatment episodes). WT refers to wild-type EcN. SynCon1 consists of a mixture of three bacterial strains, each carrying one of inducible promoters that control the expression of hemolysin. In comparison to SynCon1, SynCon2 includes three therapeutic strains with additional XOR Switch. In SynCon3, the three bacterial strains carry sensing module II to achieve bacterial lysis. **c** Average body weight changes of each group mice were measured weekly. **d** Rectal bleeding scores were evaluated by hemoccult testing during treatment duration. **e** Fecal consistency of AOM/DSS model mice treated with different regimens. **f** Kaplan-Meier survival curves of mice in different groups. **g** Total number of polyps and **h** Colon length of each group were tested after 68 days (One-way ANOVA with Tukey post-test; *p < 0.05 and **** *p* < 0.0001). **i** Macroscopic appearance of colons in AOM/DSS model mice. Black dots denote visible tumors. Scale bar (2 cm) labeled at bottom right of images. **j** Representative TUNEL staining images of the colon tissues. The distal colon of the AOM/DSS mouse model was stained using TUNEL and DAPI. TUNEL staining detected apoptotic cells, which were labeled with green fluorescence, while cell nuclei were labeled with blue fluorescence using DAPI staining. Aipathwell image analysis software was used to calculate the ratio of TUNEL-positive cells, and the result was labeled in the right corner of the image. The upper images display the scanned results of colon sections. The red box denotes the enlarged area and is presented in the lower image. Within the magnified image, the colon’s muscle layer is situated on the left, while the mucosal layer is on the right, with boundaries delineated by dashed white line.

To explore the feasibility for applying designed circuit in the in situ tumor context, we examined the efficacy of programmable bacteria in an AOM/DSS induced colitis-related mouse CRC model (Fig. 5a). Since a mixture of engineered strains containing different payloads showed synergistic therapeutic efficacy in mouse models of CRC liver metastasis^28^, three synthetic bacterial consortiums (SynCon) were tested. SynCon1 comprises three strains, wherein each strain harbors one of the inducible promoters and regulates hemolysin expression. In contrast to SynCon1, the three therapeutic strains of SynCon2 incorporated XOR Switch through the sensing module I (Fig. 1a). The constituents of SynCon3 bear sensing module II and XOR Switch, facilitating both signal amplification and cellular lysis. Oral administration of EcNs effectively colonized mice’s intestinal tract, as evidenced by a stable population ranging from 1% to 3% (Fig. 5b, Supplementary Figs. 15a and 16). SynCon displayed an approximately equal distribution of its constituent members, accounting for about 12-15% of the total EcN (Supplementary Figs. 15b). Moreover, the recombinant plasmids based on pSB1A3 exhibited a retention rate of approximately 40% within the mouse gastrointestinal tract, whereas those based on pSB4C5 demonstrated a retention rate of around 30% (Supplementary Fig. 17a). A sharp decrease in the bacterial population was also observed during each DSS treatment (Fig. 5b). Although AOM/DSS-induced mice from PBS, WT and pLldR-*hlyE* groups experienced significant weight loss, the administration of SynCon partially restored their weights (Fig. 5c and Supplementary Fig. 18). Compared to PBS group, EcN treatment not only relieved symptoms of severe bleeding and perianal bleeding (Fig. 5d and Supplementary Fig. 19a), but also improved fecal inconsistency induced by AOM/DSS (Fig. 5e and Supplementary Fig. 20). In contrast to the single strain pLldR-*hlyE*, SynCons intervention significantly reduced the number of polyps (Supplementary Fig. 21a). In terms of colon length, only SynCon2 showed a significant increase in colon length (*p* = 0.001, One-way ANOVA with Tukey post-test, Supplementary Fig. 21b). Although no differences were observed in occult blood score (Supplementary Fig. 21c), the average fecal consistency score in the SynCon intervention group was lower than that in the pLldR-*hlyE* group (Supplementary Fig. 21d) during the last two weeks.

Kaplan-Meier survival curves also showed that the SynCons group increased the survival rate of AOM/DSS treated mice (Fig. 5f). However, the survival rate of 5-FU group in phase I was dramatically decreased compared to Ctrl group (Supplementary Fig. 19b, p = 0.012, Log-rank test). Furthermore, the mortality rate of the PBS group reached 30% (Fig. 5f). The number of colon polypoid tumors in SynCon1, SynCon2 and SynCon3 was reduced by 24%, 48% and 40% compared with PBS group, respectively (Fig. 5g). Among them, SynCon2 showed a comparable number of polyps with 5-FU group, slightly lower than that of plac-*hlyE* (Supplementary Fig. 19d). SynCon2 and SynCon3 groups also increased the colon length of model mice compared with the PBS group (Fig. 5h, i, Supplementary Fig. 22). Hematoxylin and eosin (H&E) staining showed that the colon section from the Ctrl group displayed intact surface epithelium, intestinal glands, stroma, and submucosal layer (Supplementary Fig. 23). In contrast, the PBS group mice showed infiltrating inflammatory cells and atrophied crypts. Meanwhile, probiotic intervention reduced the colonic developmental abnormalities and structural damage. While local crypt atrophy and loss were still observed in the WT, plac*-hlyE*, and SynCon1 groups, the structural characteristics of SynCon2 and SynCon3 resembled those of the Ctrl group. However, 5-FU treatment did not show evidence of further improvement in inflammatory cell infiltration. Distinct from the lower pro-inflammatory cytokines in SynCons, plac*-hlyE* treated mice exhibited higher serum levels of LPS, TNF-α, IL-6, and IL-1β (Supplementary Fig. 24). Their mRNA levels of these inflammatory agents in colon tissue revealed similar trends (Supplementary Fig. 25), suggesting the potential oral risks of constitutive expression of the therapeutic strain. In addition, SynCons inhibited tumor proliferation and improved intestinal barrier via regulating the expression levels of tumor proliferation markers (Supplementary Fig. 26) and intestinal barrier-related proteins (Supplementary Fig. 27). Loss of control over cell apoptosis can allow cancer cells to survive for a longer period^53^. ELISA analysis showed that compared to the PBS group, SynCons intervention increased the levels of the tumoral apoptosis factors p53 and Bcl-2 associated X protein (Bax) and decreased the expression level of the anti-apoptotic factor B-cell lymphoma 2 (Bcl-2) (Supplementary Fig. 28). TUNEL staining results also demonstrated the SynCons enhance the apoptosis level of tumor cells (Fig. 5j). Collectively, SynCon2 has the best therapeutic efficacy and safety profile compared to the high mortality of 5-FU and the high inflammation level of plac-*hlyE*.

### Synthetic bacterial consortiums modulate AOM/DSS-induced gut microbiota dysbiosis

**Fig. 6:**
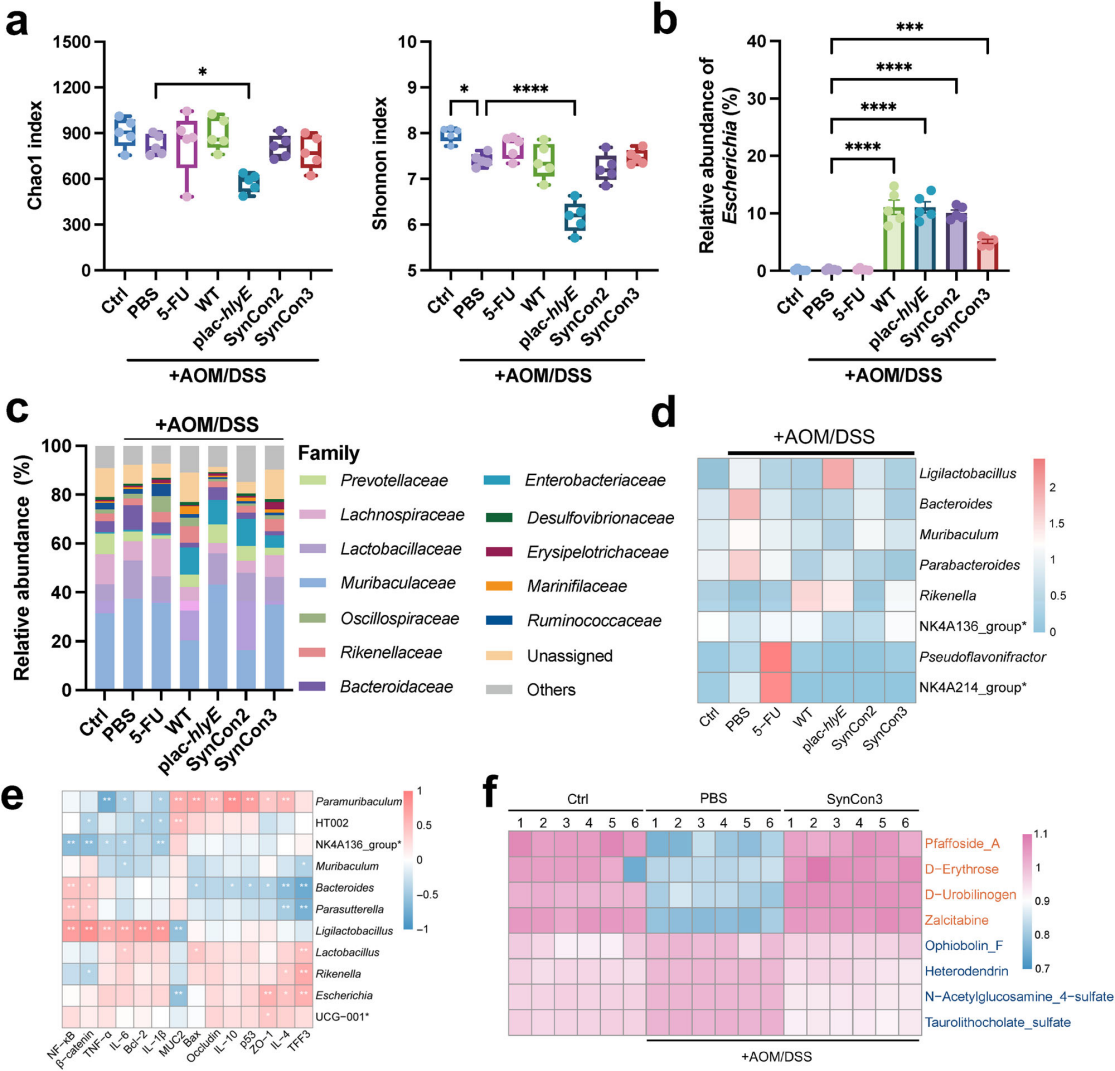
Gut microbiota and metabolites analyses of AOM/DSS induced CRC mice treated with synthetic bacterial consortium. **a** The alterations in alpha diversity indices of gut microbiota were assessed, with species richness and evenness represented by the Chao1 and Shannon indices, respectively (*n* = 5, ± s.e.m; One-way ANOVA with Tukey post-test; *p < 0.05, **** *p* < 0.0001). **b** Relative abundance of *Escherichia* (*n* = 5, ± s.e.m; One-way ANOVA with Tukey post-test; ***** *p* < 0.0001). **c** Relative abundance of microbial composition at family level. Proportions are the average of five samples. **d** At the genus level, the heatmap exhibited the differences in gut microbiota observed among the groups. **e** Spearman’s correlation analysis of microbiota and physiological indexes. Correlations with adjusted *p* < 0.05 by the Benjamini-Hochberg FDR method are marked with * symbols. **f** Heatmaps of the differential metabolites between Ctrl, PBS and SynCon3 group. NK4A214_group*: Ruminococcus_NK4A214_group; NK4A136_group*: *Lachnospiraceae*_NK4A136_group; UCG-001*: *Prevotellaceae*_UCG_001.

The possible effect of SynCon2 and SynCon3 on intestinal microbiota was further investigated in light of the beneficial therapeutic outcomes for CRC treatment. In addition to the decline in Chao index and Shannon index of plac-*hlyE* group, the α-diversity in the SynCons and 5-FU was considerable with Ctrl group (Fig. 6a). The administration of either a single EcN strain or SynCons significantly enhanced the relative abundance of *Escherichia*, with colonization rates of 5% colonization for SynCon3 and 10% for other groups (Fig. 6b). The dominant families were *Lactobacillaceae*, *Muribaculaceae*, *Lachnospiraceae* and *Prevotellaceae* (Fig. 6c). Likewise, a significant increase in *Enterobacteriaceae* was observed in the EcN treatment group. Compared to the PBS group, all EcN intervention groups showed an increase in the abundance of *Lactobacillus*, *Rikenella*, and *Clostridia*_vadinBB60_group (Supplementary Fig. 29). *Lactobacillus* is commonly regarded as a beneficial bacterium^54^, while *Clostridia* are a predominant group for butyrate production^55^. Additionally, the WT group exhibited an upregulation in the abundance of *Alistipes*, *Rikenellaceae*_RC9_gut_group, and *Odoribacter*. SynCon2 exhibited an increase in the abundance of *Prevotellaceae*_UCG-001. SynCon3 enhanced the abundance of *Akkermansia*, *Alistipes*, *Odoribacter*, and *Paramuribaculum*. *Akkermansia muciniphila* can metabolize mucin and is postulated as a prospective probiotic^56^. *Rikenellaceae*_RC9_gut_group^57^, Alistipes^58^, *Odoribacter*^59^, and *Rikenella*^60^ are considered short-chain fatty acids producers. Linear discriminant analysis Effect Size (LEfSe)^61^ indicated that *Ligilactobacillus*, *Bacteroides*, *Muribaculum* were the most significant characteristics of the PBS group, while *Lachnospiraceae*_NK4A136_group and *Rikenella* decreased significantly compared with Ctrl group (Fig. 6d and Supplementary Table 1). The SynCon intervention groups contributed to restore the abundance of these genera (Supplementary Fig. 30).

Paired spearman rank correlation coefficients were employed to identify interactions among members of the intestinal consortium and visualized as heat map (Supplementary Fig. 31). The diagram reveals two competing clusters: one composed of *Rikenella* and *Lactobacillus*, and one composed of *Muribaculum*, *Bacteroides* and *Parasutteralla*. In addition, *Lachnospiraceae*_NK4A136_group was negatively correlated with *Prevotellaceae*_UCG-001, *Escherichia* and *Ligilactobacillus*. Spearman’s correlation analysis of gut microbiota and physiological indexes showed that *Ligilactobacillus* was positively correlated with tumor proliferating factors NF-κB and β-catenin, anti-apoptotic marker Bcl-2, inflammatory factors IL-1β, IL-6 and TNF-α, and negatively correlated with gut barrier related protein MUC2. *Bacteroides*, on the other hand, was positively correlated with NF-κB and β-catenin, while negatively correlated with pro-apoptosis marker p53, Bax, anti-inflammatory related cytokines IL-10 and IL-4, and gut barrier related proteins TFF3 and ZO-1. In contrast, *Lachnospiraceae*_NK4A136_group was negatively correlated with NF-κB and β-catenin, IL-1β, IL-6, and TNF-α (Fig. 6e). Compared to the PBS group, the Ctrl group exhibited a downregulation of 2,647 metabolites and an upregulation of 3,666 metabolites (Supplementary Fig. 32a). Relative to the PBS group, 4,066 metabolites were upregulated in the SynCon3 group, while 3,281 metabolites were downregulated (Supplementary Fig. 32b). Both Ctrl vs. PBS and PBS vs. SynCon3 comparisons showed differential metabolites enriched in the glycerophospholipid and linoleic acid pathways (Supplementary Fig. 33). Compared to the Ctrl group, the PBS group showed increased abundance of Pfaffoside_A, Tetrahydrocortisone, D-Erythrose, D-Urobilinogen, and Zalcitabine (as visualized in Heatmap A) and decreased abundance of Ophiobolin_F, Heterodendrin, N-Acetylglucosamine_4-sulfate, Taurolithocholate_sulfate, and Belladine (Supplementary Fig. 34a). Compared to the PBS group, the SynCon3 intervention resulted in elevated abundance of Ammoresinol, Delcosine, Leucomycin_A6, N-Oleoyl_dopamine, and alpha-Phocaecholic_acid, and a reduction in the abundance of Methyl_acetyl_ricinoleate, alpha-Ergocryptine, 1,2-Didecanoylglycerol, Leukotriene_F4, and Mupirocin (Supplementary Fig. 34b). The top 5 differential metabolites were examined among three groups. Further analysis of the top 5 differential metabolites showed that SynCon3 supplementation reversed the changes in Pfaffoside_A, D-Erythrose, D-Urobilinogen, Zalcitabine, Ophiobolin_F, Heterodendrin, N-Acetylglucosamine_4-sulfate, and Taurolithocholate_sulfate observed in the PBS group (Fig. 6f and Supplementary Fig. 35).

## Discussion

Due to the Warburg effect, a large amount of lactate is released into the TME, leading to a decrease in pH. On the other hand, the rapid tumor proliferation results in a depletion of oxygen^39^. Therefore, lactate, pH, and hypoxic-inducible promoters pLldR, pCadC, and pPepT were chosen as the sensing elements for the engineered strains to distinguish unique organ environments. The expression of mRFP by pPepT is exclusively observed in anoxic environments. Similarly, gene circuits reliant on pPepT are triggered when oxygen levels plummeting below 0.5%, thus constraining the dissemination of *Salmonella bacteria* from organs^45^. Transcriptional activation of pLldR and pCadC was observed under 10 mM lactate and pH 5.3, as reported by Chien et al., resulted in a remarkable reduction in the escape rate of *E. coli* from solid tumors, from 10^0^ to 10^−3^ and 10^−2^, respectively^39^. Serine integrases based amplification gene switches have been used in the design of biosensing systems^62^. These genetic devices enable bacteria biosensors to achieve reliable detection, multiplex logic, and signal amplification^63^. Consistent with our study, the amplifying gene switch magnified the output signals of NO and glucose promoters by 1.5 to 2.6-fold^64^. By introducing different replication initiation sites (Ori) and antibiotic resistance genes, we constructed a dual-plasmid system harboring sensing module and therapeutic module separately. Such system can avoid crosstalk or competition of different modules, thereby improving its stability and reliability^65,66^. The established mathematical model can accurately describe the fundamental characteristics and dynamics of TME-induced engineered bacterial strains (Fig. 2). The validated model can be used for predictions under various conditions and indirectly forecast the expression levels of therapeutic proteins in the engineered strains^28^. This, in turn, provides guidance for subsequent therapeutic strategies at the animal level. Furthermore, the mathematical model employed in this study holds promise as a reference for the design of similar biological systems.

The potential protein payload must be released from the bacterial vector and possesses high cytotoxicity against malignant cancer cells. HlyE is a small hemolysin exported from *E.coli* via extravascular vesicle^67^, which has been widely used for cancer therapy due to its high cytotoxic activity^48,68^. In vitro co-culture confirmed that wild-type EcN had no effect on the viability of CRC cells. Under induction conditions, EcNs modified with environmental response promoters allowed for ex vivo hemolysin release. Cell death efficiency was positively correlated with bacteria population size and exposure time, which was consistent with the amplitude of payload release. Bacterial lysis diminishes the cytotoxicity of genetically modified EcNs, which can be ascribed to the decline in population and effective payload content. As a living drug, programmable EcNs exhibited autonomous control in subcutaneous tumor model, resulting in approximately 60% inhibition of tumor growth via sensing TME and releasing hemolysin. Recent finding revealed that acid promoter adiA can regulate the expression of cytolysin, supporting approximately 79% of tumor regression^69^. The scalability of payload capacity can enhance the therapeutic efficacy of engineered EcN based platform. Given that most proteins are not readily secreted by EcNs^70,71^, many efforts have been directed towards relying on secretory tags to translocate recombinant proteins from the cytoplasm^29^. However, the compatibility of secretory tags and payloads remains a long-standing challenge^72^. *φ*X174E has been employed in multiple studies^47,73^, and developed for bacterial host lysis and in vivo drug delivery^28^. For example, synchronous lysis circuits mediated by quorum sensing allowed bacteria to release different payloads of hemolysin, immune factors and apoptotic peptides^28^. In another example, attenuated strains of *S. typhimurium* were designed to self-destruct using *φ*X174E when sensing an invasion of tumor cells, thereby reducing 2.3 fold tumor load^74^. Parallel to the aforementioned studies, the TME-regulated *φ*X174E facilitated tumor regression of approximately 45%-50% in subcutaneous tumor models through the release of CCL21 and CCD.

SynCons based on cell interaction programming have shown promise and potential applications in human health supervision^75–77^. Oral inoculation of 17 *Clostridium* strains in mouse models enhanced Treg cell abundance and attenuated colitis and allergic diarrhea^78^. A population control circuit consisting of three strains was implemented to release therapeutic payloads in TME, demonstrating 1.6-4.0 times greater inhibition of subcutaneous tumors in mice than that of single strains^28^. Our study also observed that SynCons exhibited superior therapeutic efficacy in the AOMDSS model as compared to single strain. This could be attributed to the existence of multiple specific promoters in SynCons, which enhances the likelihood of triggering expression. Moreover, additional XOR Switch has also contributed to the amelioration of CRC. Within the gastrointestinal milieu, the proportionate distribution of SynCon members aligned with their in vitro compositions, thus culminating in optimal therapeutic efficacy through synergistic interactions. Notably, the effectiveness of oral probiotics was considerably lower than that of intratumoral administration (Figs. 4 and 5). This discrepancy may be attributed to the difficulty of reaching the lesion site. Oral administration of unshielded bacteria are prone to destruction by environmental factors such as stomach acid and the intestinal milieu^79^. Encapsulating bacteria with alginate-chitosan-alginate (ACA) has been shown to enhance their activity in gastrointestinal tract^49,80^. In the complex microbial environment of the intestine, plasmid stability in engineered bacteria is a key issue. The plasmid retention rate of the engineered EcN in this study was about 45% (Supplementary Fig. 15b). This loss of plasmid might be due to the expression burden of the synthetic circuit^81^. *L. paracasei* BL23 only retained 35-60% plasmid in the rat intestine^82^. Danino et al. also reported plasmid loss in *S. typhimurium*^83^. To minimize plasmid loss in vivo without antibiotic selection, studies have utilized plasmid stabilization systems, such as *hok*/*sok*^84^ and *alp7* cassettes^28^, or integrated genetic circuits into the host genome^85^. Considering the smaller metabolic burden, the *hok*/*sok* cassette was tested. The loss rate of pSB1A3 skeleton plasmid was reduced from 60% to 20% in the intestinal environment after integrating this cassette (Supplementary Fig. 16b). Considering the observed EcN escape in liver and spleen (Supplementary Fig. 14), bacterial chemotaxis towards tumor cells should also be further considered. To this end, the specific adhesion effect of HlpA expressing EcN on RKO and SW480 cells was verified (Supplementary Fig. 36)^29^. As previously reported, 5-FU exhibited promising therapeutic efficacy while also demonstrating severe toxicity^26^. One theory is that standard chemotherapy is used in the vascularized area, bacteria act synergistically in the ischemic tumor compartment^86^. Therefore, the interaction between programmed SynCons and chemotherapeutic agents needs to be further explored. The addition of *φ*X174E reduced the efficacy of SynCons against CRC, possibly attributed to a reduced population and effective payload concentration^87^. Nevertheless, lysis genes can enable the transient release of therapeutics and rapid elimination of bacterial delivery agents upon completion of treatment, thereby minimizing potential host side effects^88^. For example, *Salmonella*-based drug delivery system can activate flhDC to drive cell invasion and induce lysis to release therapeutic proteins into tumor cells^74^. Given these findings, future efforts should focus on fine-tuning the expression of lysis genes to precisely control the spatiotemporal release of therapeutic agents in vivo. Some genetic circuits, such as focused ultrasound based^63^ or near-infrared light-sensitive based non-invasive control elements^89^, as well as protein degradation element SsrA^90^, may offer solutions to achieve this delicate balance.

CRC models induced by AOM/DSS exhibit perturbed gut microbiota structure^49^. The SynCons modulated the α-diversity of gut microbiota structure by promoting gut remediation. The observed improvements in body weight, survival, and colon length among the probiotic-treated mice lend support to this notion. Growing attention has been received to the mutual modification between microbiota and chemotherapeutic drugs^91,92^. Compared with PBS, 5-FU reduced *Ligilactobacillus* and *Bacteroides*, while increasing *Pseudoflavonifractor*^93^. In addition to its anti-cancer effects, the rapid weight loss, decreased survival, and shortened colon length in the 5-FU treatment group were consistent with previous studies, indicating the ambivalent nature of chemotherapy^26,94^. In the AOM/DSS model, LEfSe results between groups solicit specific microbial taxa. Consistent with previous studies, AOM/DSS treatment was associated with increased abundance of *Bacteroides*^95–97^, *Muribaculum*^96,97^, and *Parabacteroides*^97^, while *Lachnospiraceae*_NK4A136_group^96^ and *Rnikenella*^97^ showed decreased abundance. Of these taxa, the presence of *Bacteroides* is known to be positively correlated with intestinal inflammation and the occurrence of colorectal cancer^98,99^. The decrease of butyrate-producing bacteria *Lachnospiraceae*_NK4A136_group^100^ in the PBS group may compromise intestinal barrier integrity and increase the risk of intestinal inflammation^49,101^. Conversely, the SynCon2 and SynCon3 facilitated the growth of *Lachnospiraceae*_NK4A136_group while reducing the abundance of *Bacteroides*. Moreover, Wang et al. reported a positive correlation between *Bacteroides* and the inflammatory cytokines TNF-α and IL-1β, while *Lachnospiraceae*_NK4A136 was negatively correlated with these two markers^95^. This further elucidates the negative or positive roles of *Lachnospiraceae*_NK4A136 and *Bacteroides* in the development of CRC.

Radiotherapy, chemotherapy, or their combination can induce various adverse effects^102^, such as gastrointestinal and neurotoxicity. Probiotics offer a minimally invasive approach by restoring gut microbiota dysbiosis during CRC therapy. However, the efficacy of natural probiotics as therapeutic agents remains limited. A crucial design of this biotherapeutic system is coupling of TME response promoters and therapeutic payloads expression, which generates more precise organotropism and extends the safety of bacteria-based platforms in precision therapy. However, the dual-plasmid system contains redundant elements unrelated to the therapeutic target. In the future, integrating them into a single plasmid or incorporating them into the bacterial genome using CRISPR may enhance the stability and reliability of engineered bacteria in clinical settings. The unintended release of engineered bacteria outside the designated environment should also be prevented by introducing lethal genes. Nonetheless, the U.S. Food and Drug Administration (FDA) permits the use of investigational drugs obtained outside of clinical trials in emergency situations^103^. This study may offer an additional option for patients with advanced stage colorectal cancer.

## Conclusion

We designed an EcN-based platform for CRC therapy, whereby the TME triggers the expression of payloads. In vitro experiments demonstrated that the XOR Switch augmented the output of the TME response promoter, enabling therapeutic strains to effectively suppress the activity of CRC cells. By virtue of their facultative anaerobic nature and the controlled expression of therapeutic payload, genetically encoded EcNs inhibited subcutaneous tumor growth without significant adverse effects. In the AOM/DSS model, SynCons harboring multiplexed biosensors exhibited improved efficacy and avoided off-target toxicity. Although bacterial therapies are still in the preliminary stage of research, the approach described here could enhance the use of bacteria as a sophisticated diagnostic device, thereby improving current oncology therapies. For example, we envision using Boolean logic gates to achieve more precise activation, which can distinguish microenvironments of various tumor type^64^. As the advancement of biomedical engineering technologies, engineered probiotics are poised to serve as a precise and robust tool for desired human disease, offering a safe and effective means for topical treatment regimens targeting focal sites.

## Methods

### Host strains and culturing

*E. coli* Nissle 1917 was purchased from Biobw (China, Beijing) and *E. coli* DH5α was previously stored in lab. All bacteria were cultured in LB broth containing appropriate antibiotics (100 μg mL^−1^ ampicillin, 50 μg mL^−1^ kanamycin, and 10 μg mL^−1^ tetracycline) at 37 °C.

### Plasmids and engineered bacteria library construction

The recombinant plasmids were verified by sequencing and then transformed into EcN to construct the engineered strains (Supplementary Table 2-4 and Supplemental Methods: *Molecular biology*).

Mathematical model. Ordinary differential equations based on hill equation and logistic equation were used to describe the fluorescence changes and bacterial growth of the biosensor. Specific equations and parameters can be found in Supplemental Methods: *Modeling*.

### Protein quantitative analysis

Protein expression levels in colon and serum samples were analyzed using enzyme-linked immunosorbent assay (ELISA) kits. For detailed information on the assay kit and the detected biomarkers, please refer to Supplemental Methods: *Biomarker analysis*.

### Western blot analysis

Mouse primary antibody against His-Tag and HRP-conjugated Goat Anti-Mouse IgG secondary antibody were utilized to detect protein expression in engineered strains within tumor tissues (Supplemental Methods: *Protein expression analysis*).

### Characterization of biosensor strains in vitro

Each variant strain was cultured in LB medium overnight with appropriate antibiotic (37 °C, 150 rpm), and then used for induction experiments in the next morning. The cultures of variant strain were transferred to 96-well plates (3 replicates) and diluted with 125 μL LB broth to OD_600_ = 0.1. As for lactate, pH and hypoxic biosensor assays, the corresponding EcNs were detected in LB medium with different L-lactate (Sigma-Aldrich) concentrations (0, 0.1, 1, 5, and 10 mM), pH values (5.5, 5.8, 6.3 and 7.3), and oxygen conditions (normoxic, 20%；anoxic, 0%) at 37°C for overnight incubation, respectively. To realize anoxic condition, the tested EcNs were statically cultured in anaerobic bags containing oxygen indicator (Hopebiol, China). After 16-20 h of growth, their absorbance (OD_600_) and mRFP fluorescence intensity (excitation λ: 584 nm/10 nm, emission λ: 607/10 nm) data were measured using a microplate reader (Thermo Fisher Scientific, USA). After subtraction of background fluorescence signal (untransformed EcN), the normalized fluorescence of biosensor strains was obtained by dividing raw mRFP pixel intensity by OD_600_ value. All triplicate values were averaged. To capture fluorescent images, Olympus BX53 (40 x) was used to observe and photograph the biosensor bacteria after incubation for 12 hours under induced (10 mM lactate; pH 7.3; 0% O_2_) or non-induced conditions.

### Cell culture

CT-26 mouse colon cancer cell, RKO human colon adenocarcinoma cell, and SW480 human colon cancer cell were purchased from Procell (Wuhan, China). All cells were cultured at 37 °C with 5% CO_2_ in DMEM (Basal Media, Shanghai, China), supplemented with 10% fetal bovine serum (Gemini, USA), and 1% penicillin/streptomycin (Gibco, USA).

### Biosensor strains were tested in supernatant of cell cultures

All cell lines were inoculated in 25 cm^2^ culture bottle containing 6 mL complete medium (6 replicates) with an initial cell count of 10^5^. For the next 5 days, the cell cultures were removed from flasks twice a day and transferred to 15 mL sterile centrifuge tubes. After centrifugation at 200 r.c.f. for 5 min, the culture medium supernatant was cryopreserved at −80 °C. Then biosensor strains were inoculated into 24-well plates containing 800 μL LB medium (supplemented with corresponding antibiotics). After that, 200 μL of stored cell cultures supernatant was thawed and incubated with the above strains. pH test paper was used to detect the pH of cell cultures supernatant. Lactate concentration of cell cultures supernatant was measured using LA Assay Kit (BC2235, Solarbio). The hypoxic biosensor was tested in a constant temperature incubator under hypoxic or normoxic condition. All bacteria were cultured for 12-16 hours, and the fluorescence and absorbance of the biosensor strains were determined using a microplate reader (Thermo Fisher Scientific, USA).

### Co-culture of therapeutic strains with colorectal cancer cells

Cancer cells were inoculated in 96-well plates containing 100 μL complete DMEM. When cancer cells adhered, 10 μL of therapeutic strains and control strains (initial OD_600_ = 0.6) were inoculated, respectively. The supernatant of the cell culture on day 3 was used as the inducer. Co-cultivation was carried out for 3 h at 37 °C^30^. After that, the media containing bacteria were removed and replaced with fresh DMEM. The cell viability of colorectal cancer cell was monitored using CCK8 kit (C0038, Beyotime, China) on the microplate reader. The Calcein/PI kit (C2015S, Biotime) was used to label live and dead cells of CT26 after different treatments. The staining results were visualized with Olympus BX53, green and red fluorescence represent live and dead cells, respectively.

### Anticancer drugs preparation

Therapeutic strains were grown overnight (37 °C, 150 r.p.m) in 5 mL LB. Then, the bacteria culture was transferred to 300 mL LB medium for expansion culture. After centrifugation at 8000 r.c.f for 10 min, the cell pellets were harvested and suspended with sterilized PBS (pH 7.4). The bacterial density was 5 × 10^9 ^c.f.u per mL for gavage and 5 × 10^7 ^c.f.u per mL for subcutaneous tumor injection^28^.

### Mouse strains and growth conditions

Female Balb/c (6-week-old, 18-22 g, for subcutaneous tumor model) and C57bL-6J mice (6-week-old, 18-22 g, for AOM/DSS induced CRC model) were purchased from Lanzhou University Animal Center. Mice were fed in a pathogen-free facility with 12/12 hours light/dark cycle and constant temperature (20 ± 3°C) and humidity (40 ± 20%) (Laboratory Animal Center of the School of Life Sciences, Lanzhou University). During study period, the animals were given free access to sterilized water and dry pellet feed. The animals were euthanized according to the study schedule or when the tumor size reached 2 cm^26^.

### Subcutaneous tumor model

CT26 colorectal cells were used to construct subcutaneous tumor mouse model. At the exponential growth stage, CT26 cells were harvested and suspended in DEME medium (no phenol red) to achieve a concentration of 5×10^7^ cell per mL. Cells were implanted at bilateral subcutaneous hind flank, with a volume of 100 μL (i.e., 5 × 10^6^ cell) per flank. Once the average tumor size reached an average of about 150 mm^3^ (i.e., 5.3 mm), the mice were randomly assigned (n = 5 for each group). Then, tumor injection was performed with 20 μL 0.9% normal saline or (i.e., 1 × 10^6 ^c.f.u) therapeutic strains at 0, 4, 7 and 11 days. Mice body weight and tumor volume were recorded every 2 days. The inhibition rate (IR%) of anticancer drugs on tumor growth was calculated as: IR (%) = (1 − T/C) × 100, where C is the average tumor weight of the control group and T is the average tumor weight of the tested drug groups^39^.

### AOM/DSS-induced CRC mouse model

On the first day of the experiment, 6-week-old C57BL-6 mice (about 18-22 g) were intraperitoneally injected with 12.5 mg/kg body weight AOM (Sigma-Aldrich). After 5 days, mice were given drinking water containing 2% (wt/vol) DSS (molecular weight 36-50 kDa, YEASEN, China) for 7 consecutive days, followed by regular drinking water for 14 days. Repeat the cycle for three times. Mice were orally given 0.9% normal saline or 5 × 10^8 ^c.f.u EcN (i.e., 100 μL bacteria suspension), five times a week for 68 days. The positive control group was intraperitoneal injected 40 mg/kg 5-FU twice a week. Rectal occult blood or gross blood, food intake and body weight of each mouse were reordered weekly. The bleeding situation was analyzed using fecal occult blood test kit (C027, Nanjing Jiancheng Bioengineering Institute, China). The negative result was scored as 0. Slight positive was scored as 1. Strong positive was scored as 2. Fecal consistency was classified as follows: 0 for normal, 1 for soft and sticky. Weight change during the study period was calculated as a percentage change in body weight relative to baseline measurements. On day 68, the mice were euthanized, and colon length and polyp number were measured.

### Biodistribution

After intratumor injection of bacteria, mice were euthanized to obtain the liver, spleen, and tumor. The harvested organs were weighed and homogenized using an automatic grinder (Tissuelyser-24L, Jingxin, Shanghai, China). Homogenates underwent sequential dilution and spread onto LB agar plates with antibiotic selection at 37 °C overnight. Colonies were enumerated and quantified as c.f.u. per gram of tissue^39^.

### Quantification of EcN population in gut microbiota

The colonization of recombinant EcN in AOM/DSS induced CRC model mice was analyzed by absolute quantitative method^104^ For experimental procedures and primer information, please refer to Supplemental Methods: *Absolute quantitative analysis* and Supplemental Table 5.

### Colon gene expression analysis

Quantitative reverse transcription PCR (qRT-PCR) was employed to quantify mRNA expression of colon genes. For experimental procedures and primer information, please refer to Supplemental Methods: *Quantitation of gene expression* and Supplemental Table 6.

### Bioluminescent assay

After 3 days of intratumoral injection of *lux*CDABE-labeled programmed EcNs, mice were anesthetized with 1% (m:v) pentobarbital sodium saline solution (Intraperitoneally, 40 mg/kg body weight)^39^. The luminous signals were measured using chemiluminescent imager (Fx6, Vilber, France) to track the number and distribution of bacteria in subcutaneous tumor^105^. During the entire imaging trial period, tumour volume was quantified to follow physical tumour growth^28^. To further determine the survival rate of probiotics in the digestive tract, C57BL/6 mice were orally given EcN labeled with *lux*CDABE (1 × 10^9 ^c.f.u), and their GI tracts were imaged with Vilber 3 h later. Bioluminescence signals in the region of interest were quantified by Image 2.1 software.

### 16 S rRNA sequencing and bioinformatics analysis

Stool samples from AOM/DSS induced CRC mice were collected and frozen at -80 °C. After the samples were slowly thawed, DNA was extracted using Stool DNA extraction kits (Omega, USA). The 16 S rRNA V3-V4 regions were amplified using specific primers and sequencing was conducted on Illumina MiSeq platform (LC-BioTechnology, China). The QIIME 2 pipeline was utilized for data processing. To determine α diversity, the Shannon index and Chao index were analyzed using Mothur v.1.31.0. LEfSe (*p* < 0.05 and logarithmic LDA score was set at 3.0) was performed using the online tool at https://www.omicstudio.cn. Heatmaps were plotted using R package version 3.6.3.

### Fecal untargeted metabolome analysis using LC-MS

50 mg of thawed faecal samples were mixed with 500 μL of pre-cooled methanol/water (1:1, v/v), homogenized with ultrasonic treatment at 4 °C for 30 min. After adding acetonitrile, the solutions were incubated at −20 °C for 1 h, then centrifuged and freeze-dried. The pre-processed samples, QC samples, and blanks were stored at −80 °C prior to LC-MS analysis. Chromatographic separations were performed using a Thermo Scientific UltiMate 3000 HPLC and mass spectrometry data were acquired using a Q-Exactive. The data was processed using Compound Discoverer 3.1.0 and metabolites were annotated based on HMDB and KEGG database. Differential metabolites were selected using two-tailed Student’s t-tests.

### Ethics Statement

The animal experimental proposal was approved and supervised by the Animal Ethics Committee of School of Life Sciences, Lanzhou University and carried out in accordance with ethical guidelines of the 1975 Declaration of Helsinki (permission number: EAF2022053).

### Statistical analysis

The study data were statistically analyzed using GraphPad Prism 8.0, and results are presented as means ± s.e.m. Statistical tests included Student’s *t*-test (two-tailed, unpaired) for two-group comparisons, One-way ANOVA with Tukey post-test for multiple-group comparisons, and Kaplan-Meier analysis with log-rank test for survivability analysis. Significance was denoted as *p < 0.05, **p < 0.01, ***p < 0.001, ****p < 0.0001.

### Reporting summary

Further information on research design is available in the Nature Research Reporting Summary linked to this article.

### Supplementary information

Supplemental Materials

Dataset 1

Reporting summary

## Data Availability

The sequencing data for 16 S rRNA have been deposited in the Sequence Read Archive (SRA) of the National Center for Biotechnology Information (NCBI) under the accession number PRJNA970984. The raw data of fecal metabolites were stored in Figshare with the 10.6084/m9.figshare.24844692.v1. All relevant data are available from the authors.
