# Supplementary material for: Enhancing tumor-specific recognition of programmable synthetic bacterial consortium for precision therapy of colorectal cancer
Source: NPJ Biofilms Microbiomes. 2024 Jan 20;10:6. doi: 10.1038/s41522-024-00479-8 (PMC10799920; doi:10.1038/s41522-024-00479-8)
Supplement: Supplementary file 1 — Supplemental Materials [file 41522_2024_479_MOESM1_ESM.pdf]

Supplemental materials for

Enhancing Tumor-Specific Recognition of Programmable Synthetic Bacterial Consortium for Precision Therapy of Colorectal Cancer

Tuoyu Zhou[1]#, Jingyuan Wu[2]#, Haibo Tang[1], Dali Liu[4], Byong-Hun Jeon[5], Weilin Jin[6], Yiqing Wang[2], Yuanzhang Zheng[7], Aman Khan[1], Huawen Han[3]*, Xiangkai Li[1]*

1. Ministry of Education Key Laboratory of Cell Activities and Stress Adaptations, School of Life Sciences, Lanzhou University, Lanzhou, China
2. Gansu Province Key Laboratory Biotherapy and Regenerative Medicine, The First Hospital of Lanzhou University, Lanzhou, China
3. State Key Laboratory of Grassland Agro-ecosystems, College of Pastoral Agricultural Science and Technology, Lanzhou University, Lanzhou, China
4. Department of Chemistry and Biochemistry, Loyola University Chicago, Chicago, Illinois, USA
5. Department of Earth Resources and Environmental Engineering, Hanyang University, Seoul, Korea
6. Medical Frontier Innovation Research Center, The First Hospital of Lanzhou University, Lanzhou, China
7. Discovery Biology, Curia Golbal Inc., New York, USA

# These authors contributed equally to this work: Tuoyu Zhou, Jingyuan Wu.

***Corresponding author:**

Prof. Xiangkai Li

E-mail: xkli@lzu.edu.cn;

Prof. Huawen Han

E-mail: hanhuawen@lzu.edu.cn.

This file includes:

I. Supplementary Methods

1. Molecular biology

2. Modeling

3. Biomarker analysis

4. Absolute quantitative analysis

5. Quantitation of gene expression

6. Protein expression analysis

II. Supplemental Figures

Supplemental Figure 1 to 36

III. Supplemental Tables

Supplemental Table 1 to 6

IV. Supplementary References

I. Supplementary Methods

1. Molecular biology

The encoded information of all encoded genes or functional DNA fragments were obtained from NCBI (https://www.ncbi.nlm.nih.gov/), iGEM Standard Biology Parts (https://parts.igem.org/) and BioCyc databases (https://biocyc.org/). In particular, the related DNA sequences of amplifying genetic logic gates were reported and annotated in previous study [1]. The DNA sequences were amplified from *E. coli* DH5α genomic DNA, plasmid pGEN-*lux*CDABE (P8781, MiaoLing, China), or synthetized by Genewiz (China) and Twist (USA, 2022 iGEM sponsor). mRFP was stored and provided in lab. All recombinant plasmids were based on pSB1A3 or pSB4C5 backbones (**Supplemental Figure 1**). pSB1A3 carries ampicillin resistant gene and a high copy replicon ColE1, while pSB4C5 carries chloramphenicol resistant gene and a low copy replicon pSC101. Amplified primers were synthesized by GENERAL Biol (Anhui, China). All recombinant plasmids were constructed using one-step cloning kit (C113, Vazyme, China) or Blunt Kination ligation kit (6127A, Takara, Japan) (**Supplemental Table 2**). All constructed plasmids were first transformed into *E. coli* DH5α for preservation and amplification, verified by DNA sequencing (Tsingke, China), and then transformed into EcN. Full information of programmable strains can

be obtained in **Supplemental Table 3** and bacteria member of synthetic consortia is listed in **Supplemental Table 4**.

2. Modeling

To describe the behavior of biosensors, we developed an ordinary differential equation describing normalized mRFP fluorescence (mRFP/cell) (y) generation in response to ambient lactate concentration (L, equation (1)), protons (H^+, equation (2)), and oxygen level (O_2, equation (3)) via XOR Switch, regulated by transcription factors LldR [2], CadC [3] and FNR [4]. For simplicity, the timescale for binding and transcription reactions is assumed much faster than that for translation [5,6]. Bacterial growth is modelled using the logistic equation with N_{max} as the maximum population size, β_N is the degradation rate and r_0 is the growth rate (equation (4)) [7]. The relationship between bacteria degradation rate (β_N) and lysis protein (L_p) can be defined as hill equation (assumed hill coefficient is 1) (equation 5) [8]. Since φX174E is placed downstream of pLldR, pCadC, and pPepT, the lysis proteins concentration (L_p)) is induced by ambient lactate (equation (6)), H^+ (equation (7)), and oxygen (equation (8)). According to equations (4) and (5), the time-dependent changes of the population of lactate (equation (9)), pH (equation (10)) and hypoxia (equation (11)) induced lysis biosensors were described. As mRFP production depends on both bacterial population and inducing signals, we can derive a formula to describe the expression of mRFP (y) in lactate induced lysis biosensors (equation (12)). Additionally, we also presented equations to describe the mRFP production in pH (equation (13)) and hypoxia (equation (14)) induced lysis biosensors. Parameter fitting was based on the dataset collected in experiments. Initial values for parameters were set using reported values [8,9], and optimization of parameters was performed using the least squares analysis based on the Levenberg-Marquardt algorithm (least_squares function, Scipy.optimize library). Specifically, a residual function, $r = y_{\text{measured data}} - y_{\text{prediction}}$, was defined to calculate the differences between model predictions and actual measurement data given the model parameters. Iterative adjustments were made to minimize the sum of squared residuals ($SSE=\sum r^2$) to ensure convergence of the fitting process.

$$\frac{d_y}{d_t} = \frac{\alpha_y K_s}{1 + K_s + \left(\frac{K_1}{1 + K_{\mathrm{L}} L}\right)} - \beta_y y \tag{1}$$

$$\frac{d_y}{d_t} = \frac{\alpha_y K_s}{1 + K_s + (\frac{K_{\mathrm{H}} + \frac{1}{\mathrm{H}^+}}{K_{\mathrm{H}} K_2})} - \beta_y y \tag{2}$$

$$\frac{d_y}{d_t} = \frac{\alpha_y K_s}{1 + K_s + (\frac{K_{\mathrm{O}_2} \mathrm{O}_2 + 1}{K_3})} - \beta_y y \tag{3}$$

$$\frac{d_N}{d_t} = r_0 (N_{max} - N) N - \beta_{\mathrm{N}} N \tag{4}$$

$$\beta_N = \frac{K_\gamma}{1 + \frac{L_0}{L_p}} \tag{5}$$

$$L_p = \frac{\alpha_y}{1 + (\frac{K_1}{1 + K_L L})} \tag{6}$$

$$L_p = \frac{\alpha_y}{1 + \frac{K_{\mathrm{H}} + \frac{1}{\mathrm{H}^+}}{K_{\mathrm{H}} K_2}} \tag{7}$$

$$L_p = \frac{\alpha_y}{1 + \frac{1 + K_{\mathrm{O}_2} \mathrm{O}_2}{K_3}} \tag{8}$$

$$\frac{d_N}{d_t} = r_0 (N_{max} - N) N - \frac{\alpha_y K_\gamma}{L_0 + \alpha_y + \left(\frac{K_1 L_0}{1 + K_L L}\right)} N \tag{9}$$

$$\frac{d_N}{d_t} = r_0 (N_{max} - N) N - \frac{\alpha_y K_\gamma}{\alpha_y + L_0 + L_0 \left(\frac{K_{\mathrm{H}} + \frac{1}{\mathrm{H}^+}}{K_{\mathrm{H}} K_2}\right)} N \tag{10}$$

$$\frac{d_N}{d_t} = r_0 (N_{max} - N) N - \frac{\alpha_y K_\gamma}{\alpha_y + L_0 + L_0 \left(\frac{1 + K_{\mathrm{O}_2} \mathrm{O}_2}{K_3}\right)} N \tag{11}$$

$$\frac{d_y}{d_t} = \frac{\alpha_y K_s}{1 + K_s + (\frac{K_1}{1 + K_{\mathrm{L}} L})} N - \beta_y y \tag{12}$$

$$\frac{d_y}{d_t} = \frac{\alpha_y K_s}{1 + K_s + (\frac{K_{\mathrm{H}} + \frac{1}{\mathrm{H}^+}}{K_{\mathrm{H}} K_2})} N - \beta_y y \tag{13}$$

$$\frac{d_y}{d_t} = \frac{\alpha_y K_s}{1 + K_s + (\frac{K_{O_2} O_2 + 1}{K_3})} N - \beta_y y \qquad (14)$$

Model parameters

α_y (production rate), 6.5-140; β_y (degradation rate), 1; K_s (TP901 biding affinity to XOR gate), 10; K_1 (LldR dimer binding affinity to pLldR promoter), 120; K_L (Lactate binding affinity to LldR dimer), 10; K_2 (CadC binding affinity to pCadC promoter), 200; K_H (H^+ interaction with CadC transcription activator), 250; K_3 (FNR dimer binding affinity to pPepT promoter), 20. K_{O_2}(O_2 binding affinity to FNR dimer), 50. N_{max} (maximum population),10; r_0 (nature growth rate), 0.5; K_γ (maximum lysis rate), 4.70-5.02; L_0 (lysis protein concentration at half the maximum lysis rate), 0.03-30.

3. Biomarker analysis

Serum samples were thawed in ice from -80 °C. Frozen colon tissues were weighed and soaked in ice-cold sterile PBS in proportion (1: 9, w/v), then homogenized using automatic tissue dissociator (Tissuelyser-24L, Jingxin, Shanghai, China) and centrifuged at 5000 g for 5 min to collect supernatant. Then, serum lipopolysaccharides (LPS; F2631, FANKEWEI, China), colon inflammatory cytokines (tumor necrosis factor-alpha (TNF-α), interleukin-1β (IL-1β), interleukin-6 (IL-6); Shanghai Enzyme-linked Biotechnology, China) and apoptosis biomarkers (P53, B-cell lymphoma 2 (Bcl-2), Bal-2 associated X protein (BAX); Shanghai Enzyme-linked Biotechnology, China) were measured using corresponding commercial kits. The total protein concentration in the homogenate supernatant was determined using a Bradford Protein Assay Kit (Beyotime, China, P0006). Absorbance was detected using Multiskan GO equipped with SkanIt Software 4.1 (Thermo Fisher Scientific, USA).

4. Absolute quantitative analysis

Briefly, stool DNA was extracted using the TIANamp fecal DNA Kit (Tiagen Biotechnology, China). Then, real-time quantitative PCR (qPCR) was performed on DNA using specific primers for total bacteria, EcN and recombinant strains. All qPCR

primer sequences are listed in **Supplemental Table 5**. The target DNA sequences were cloned to pMD-18T (Takara) and the recombinant plasmid was employed to establish qPCR standard curve. qPCR was performed on QuantStudio 5 (Applied Biosystems, USA) using TB Green Premix Ex Taq II (RR820, Takara). The gene copy numbers were calculated. All measurements were repeated three times.

5. Quantitation of gene expression

Total RNA from the colon tissue was extracted from individual homogenates with the RNAprep Pure Tissue Kit (DP431, TIANGEN Biotech) and reverse-transcribed into cDNA using the HiScript 1st Strand cDNA Synthesis kit (R111, Vazyme Biotech, China,). Then, a total of 100 ng of cDNA was mixed with TB Green Premix Ex Taq II (RR802, Takara) and quantitative reverse transcription PCR (qRT-PCR) was performed on QuantStudio 5 (Applied Biosystems, ThermoFisher, USA). The primer sequences of the target genes are listed in **Supplemental Table 6**. GAPDH gene was used as internal controls and the mRNA expression level of each gene was calculated using $2^{-\Delta\Delta Ct}$ method.

6. Protein expression analysis

Through reverse PCR and BKL kit (Takara, Japan), 6 x His-Tag was introduced at the 3 ‘end of *hlyE.* The dual-vector systems containing sensing and therapeutic elements were constructed as described. In the subcutaneous tumor mouse model, tumor samples were obtained 12 hours after administering the engineered strains via intratumoral injection. The total proteins of tumor samples were extracted by homogenization with RIPA lysis buffer provided by Total protein extraction kit (W034-1-1, Nanjing Jiancheng Bioengineering Institute). After centrifugation at 12 000g for 15 min at 4 °C, the supernatants were collected, and the protein concentrations were determined by Bradford Protein Assay Kit (Beyotime). By 10% sodium dodecyl sulfate-polyacrylamide gel electrophoresis (SDS-PAGE), proteins were separated and transferred onto a PVDF membrane (Millipore, MA, USA). The membrane was then blocked with skimmed milk (5%; TBS-Tween) for 1 hours and incubated overnight at

4 °C with specific mouse primary antibody against His-Tag (66005-1, proteintech). After washing 3 times with TBS-Tween (Beyotime, P0023C3), the membrane was incubated with second antibody (HRP-conjugated Affinipure Goat Anti-Mouse IgG (H+L), SA00001-1, proteintech) for 2 hours. The signals were visualized using the BeyoECL Plus (Beyotime, P0018S) and pictured using Fusion FX 6 (Vilber Lourmat, France).

II. Supplemental Figures

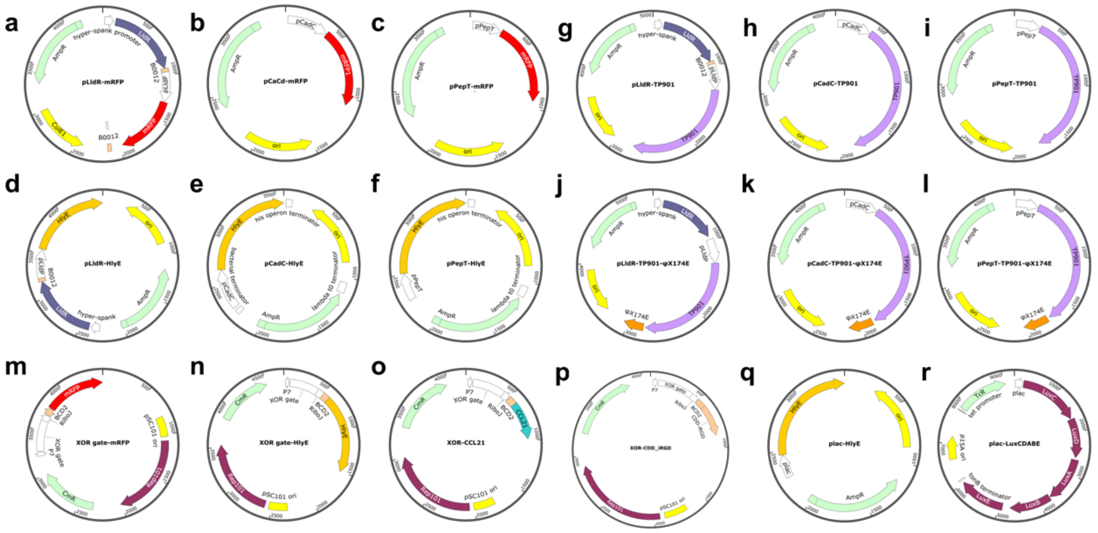

Supplemental Figure 1. Maps of plasmids used in this study. a, pLldR-mRFP. **b**, pCadC-mRFP. **c**, pPepT-mRFP. **d**, pLldR-HlyE. **e**, pCadC-HlyE. **f**, pPepT-HlyE. **g**, pLldR-TP901. **h**, pCadC-TP901. **i**, pPepT-TP901. **j**, pLldR-TP901-*φ*X174E. **k**, pCadC-TP901-*φ*X174E. **l**, pPepT-TP901-*φX174E*. **m**, pP7-XOR gate-mRFP. **n**, pP7-XOR gate-HlyE. **o**, pP7-XOR gate-CCL21. **p**, pP7-XOR gate-CDD_iRGD. **q**, plac-HlyE. **r**, plac-LuxCDABE. Among the labeled genetic elements, RiboJ is a ribozyme-based insulator, which can buffer synthetic circuits from genetic context 10,11. Besides, as a translation initial element, BCD2 can realize precise and reliable downstream gene expression 12.

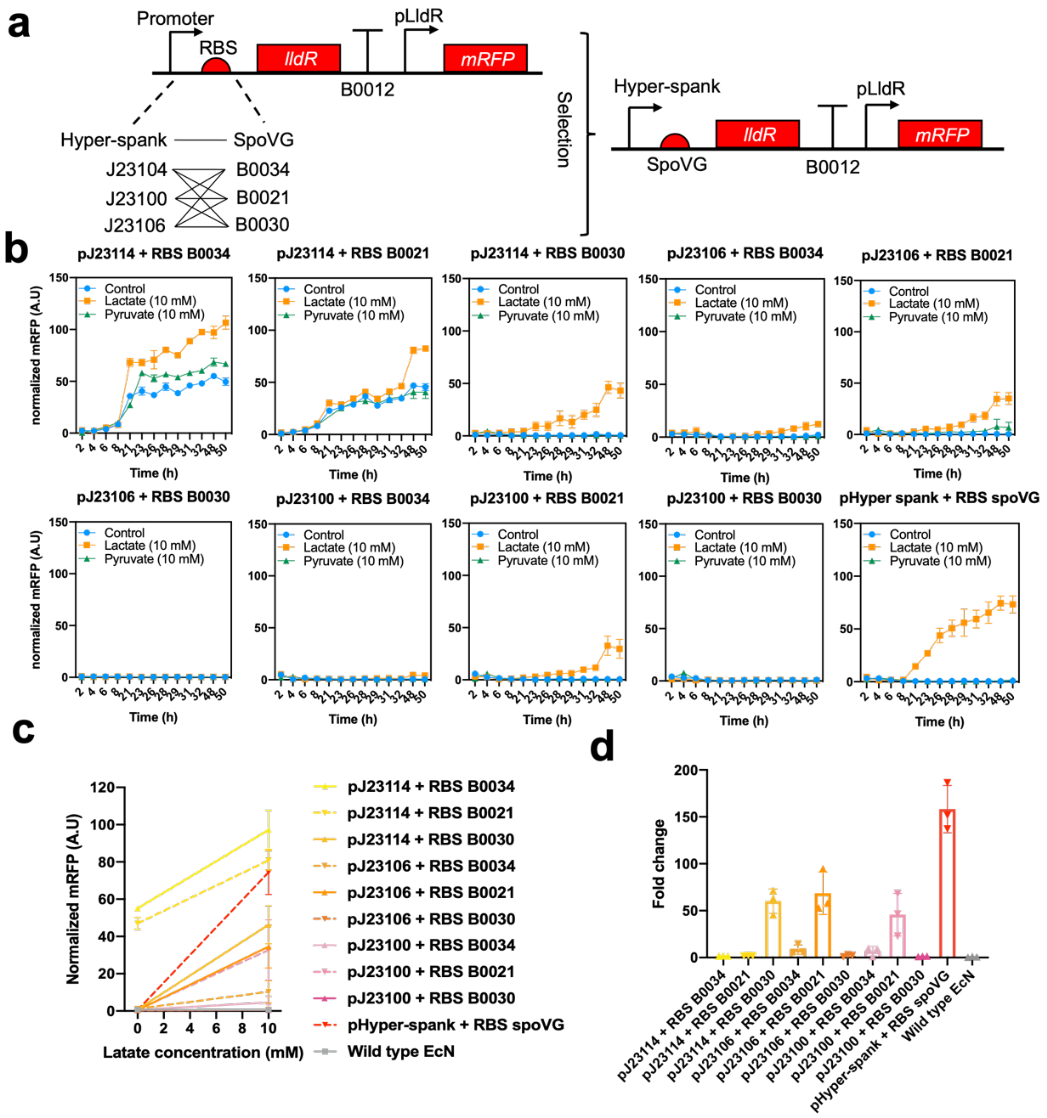

Supplemental Figure 2. ***In vitro*** **characterization of lactate biosensor variants. a**, Lactate biosensor variants were constructed by generating a library of plasmids containing various constitutive promoters driving lactate repressor and a lactate promoter driving mRFP. These plasmids were then transformed into EcN strain, resulting in 10 generated variants. **b**, Induction data from 10 different strains carrying reporter plasmid variants showed mRFP expression levels (A.U) in 0 mM and 10 mM lactate or pyruvate (n = 3, ± s.e.m). **c**, Induction data of 10 strains at 48 h. **d**, The fold-change of the lactate activator was calculated as the ratio of mRFP fluorescence in induced to non-induced states. The red dotted line in (**c**) represents the optimal strain that matched our criteria of low basal expression and high fold change upon induction.

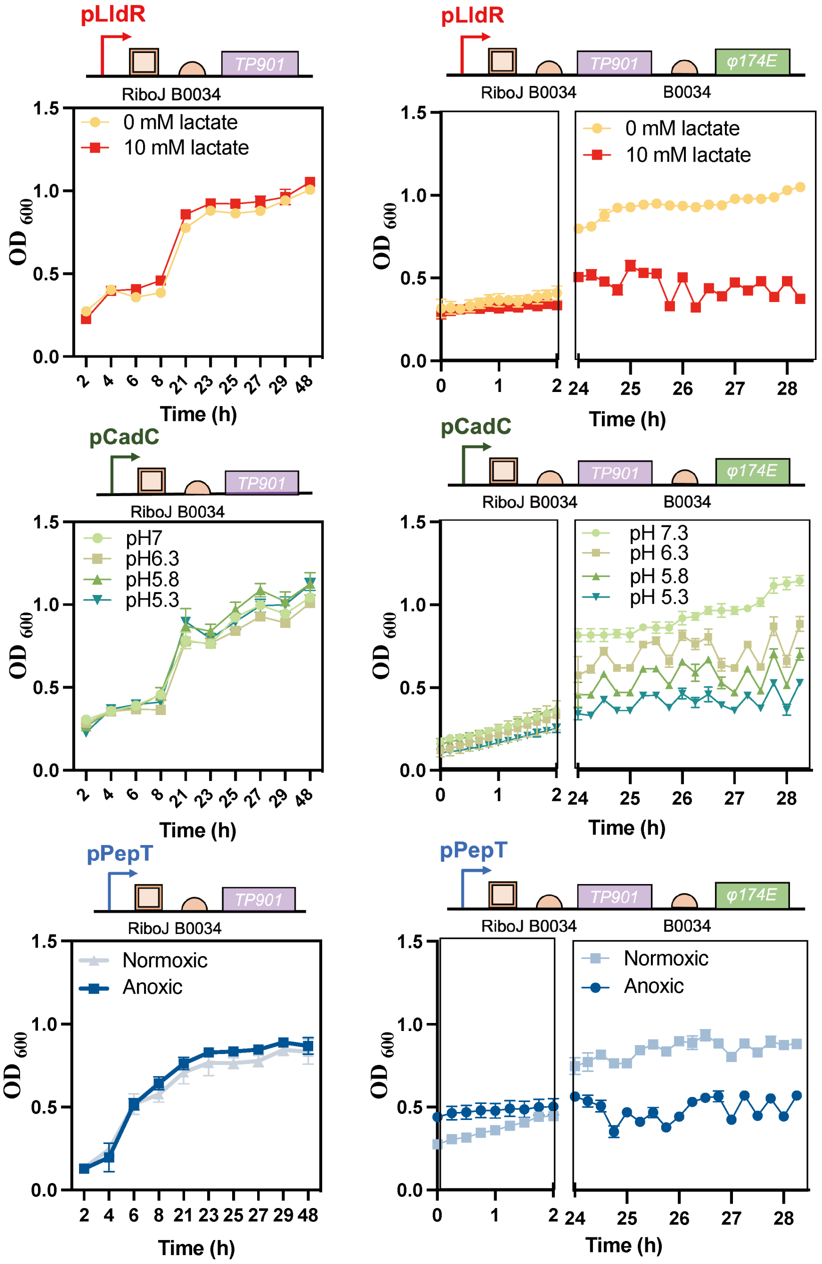

Supplemental Figure 3. Bacterial growth characteristics of biosensor variants. a-f, Up: circuit schematics of lactate, pH and hypoxia-induced biosensor strains. **a-f**, Down: optical densities measurements of bacterial cultures after growth in corresponding various concentrations of inducers. Bacterial colonies were grown at 37 ℃ in LB medium. For reporter strains shown in **a-c**, bacteria were cultured for 48 h and OD_{600} values were recorded every 1 h (n = 3, ± s.e.m). For reporters strains carrying lysis module (**d-f**), bacteria were cultured for 28 h and OD_{600} values were recorded

every 5 min (n = 4, ± s.e.m). Optical densities of the culture medium were subtracted from each measurement.

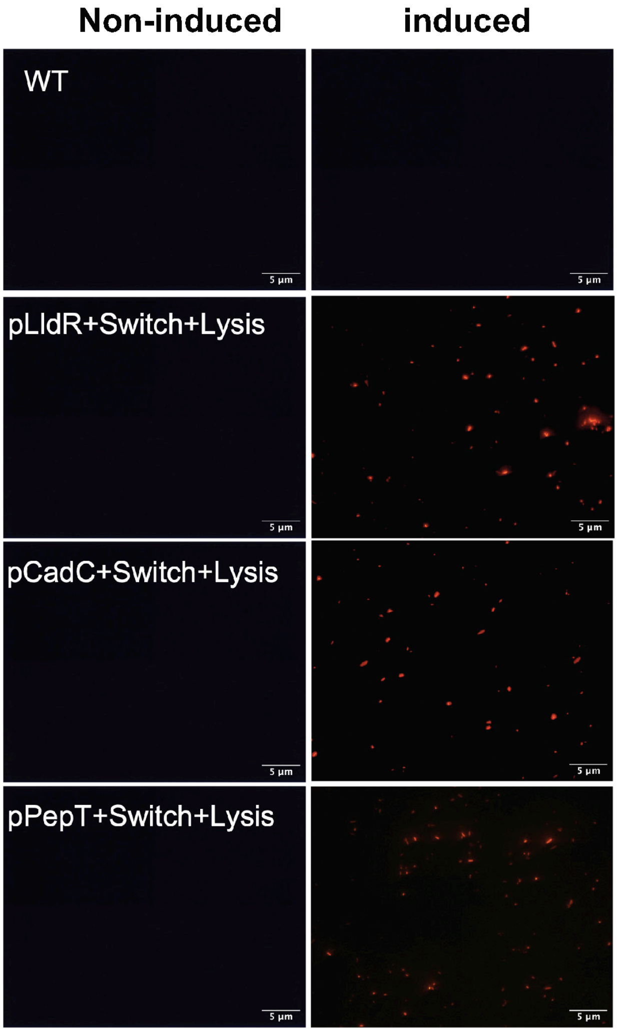

Supplemental Figure 4. Representative fluorescence image of different lysis biosensors after growing at induced or non-induced states. Bacteria were cultured in LB medium for 12 h at 37 °C. The scale bar labeled in the lower right corner of the image represents 5 μm.

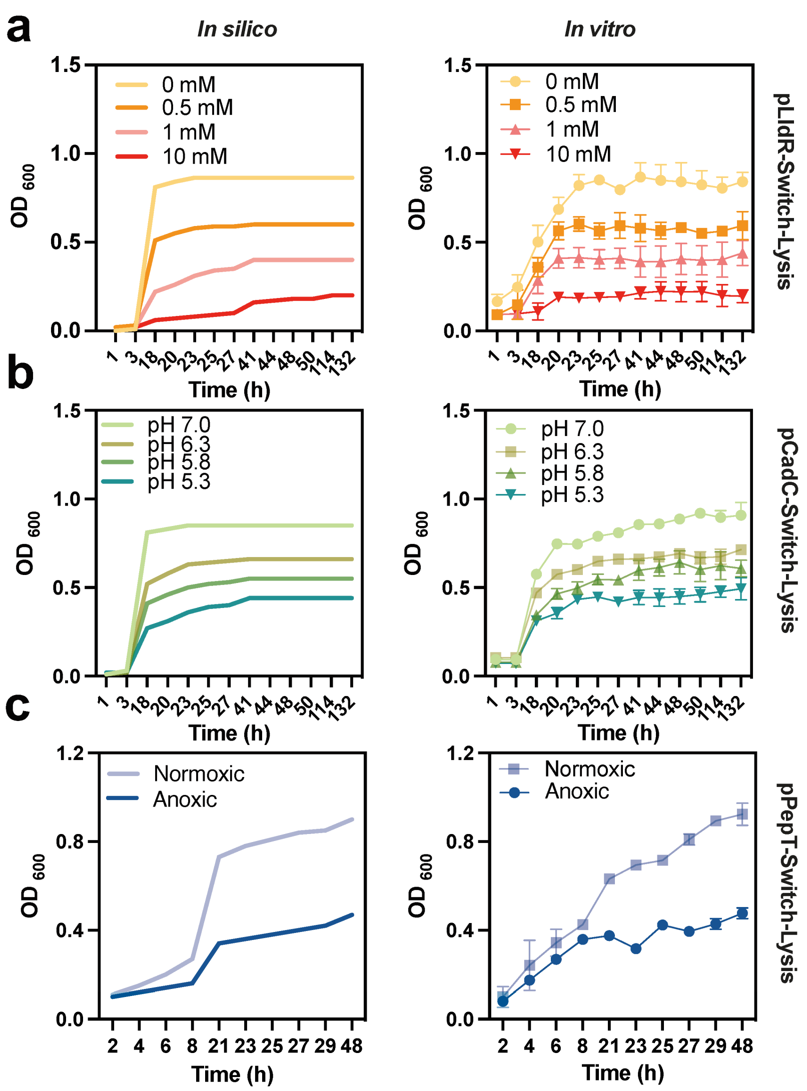

Supplemental Figure 5. Computational modelling of bacterial growth curves of lysis biosensors. Bacteria population of lactate (**a**), pH (**b**) and anoxic (**c**) induced lysis biosensors were modelled under varying environmental conditions. Modeling *in silico* predictions (Left) compared to (right) *in vitro* experimental results (n = 3, ± s.e.m).

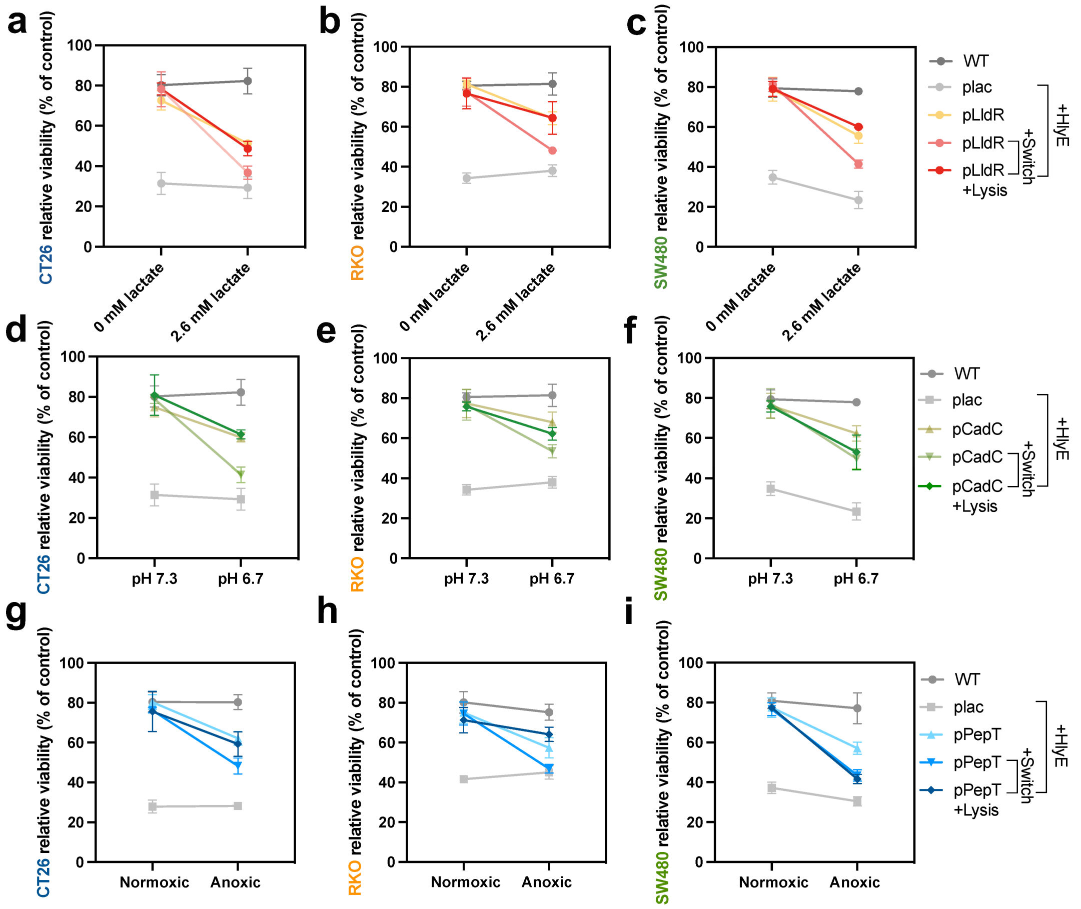

Supplemental Figure 6. Effects of lactate (**a-c**), pH (**d-e**), and hypoxia (**g-i**) induced therapeutic and control strains on the activity of CT26, RKO, and SW480. For lactate and pH-based effector strains, fresh DMEM medium was used as the non-induced condition. Cells cultured for 3 days with accumulated lactate were used as induction conditions. For the anoxic-induced engineered strain, the damage effect of the engineered strains on tumor cells was tested in anoxic and normoxic conditions respectively. Error bars indicate ± s.e.m for three measurements.

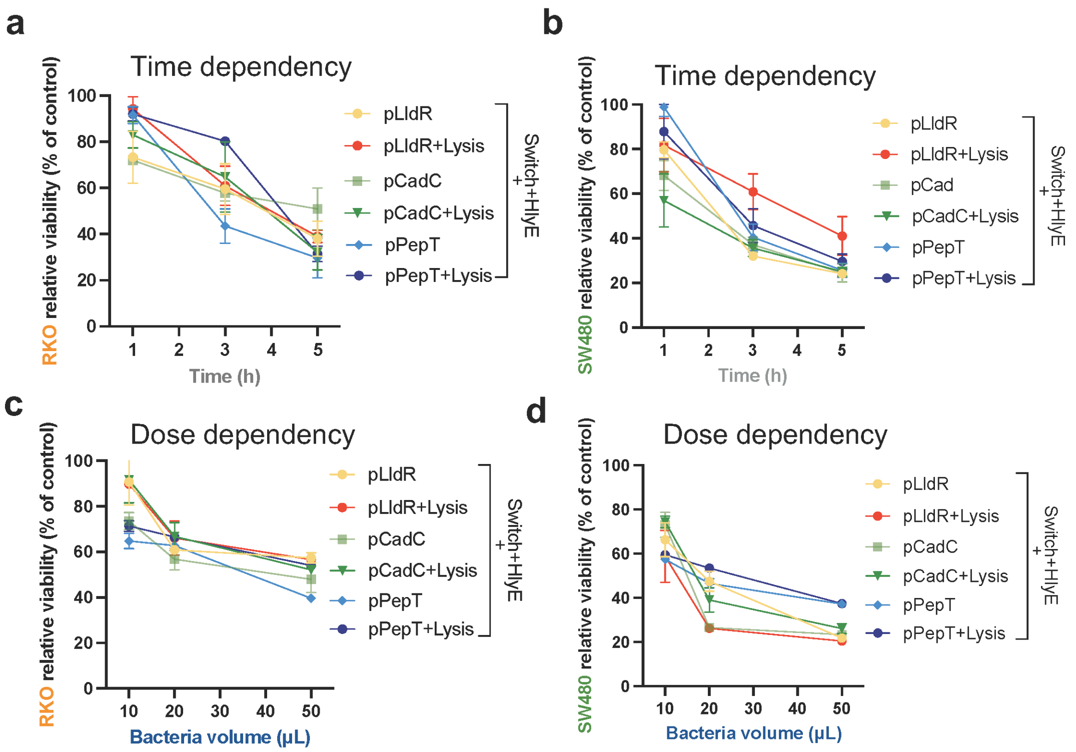

Supplemental Figure 7. a-b, RKO and SW480 viability after co-culture with 30 μL therapeutic strains (carrying *hlyE* gene) for varying time. **c-d**, RKO and SW480 cell viability after co-culture with different volume therapeutic strains over 3 h. All bacteria OD_{600} was adjusted to 0.6. Error bars indicate ± s.e.m for three measurements.

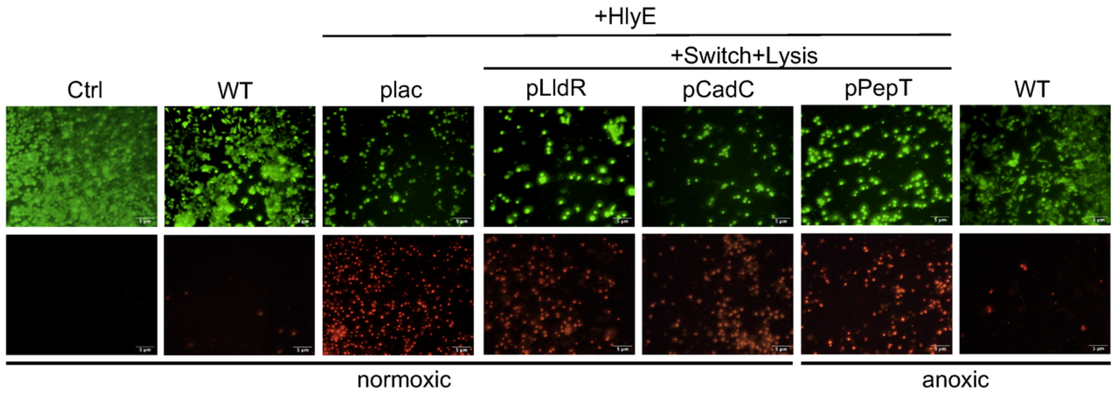

Supplemental Figure 8. Fluorescence microscope of live and dead cells after co-culture with 30 all lysis therapeutic strains for 3 h. CT26 cell line were stained with Calcein/PI (Olympus BX53, 40x magnification). The scale bar labeled in the lower right corner of the image represents 5 μm.

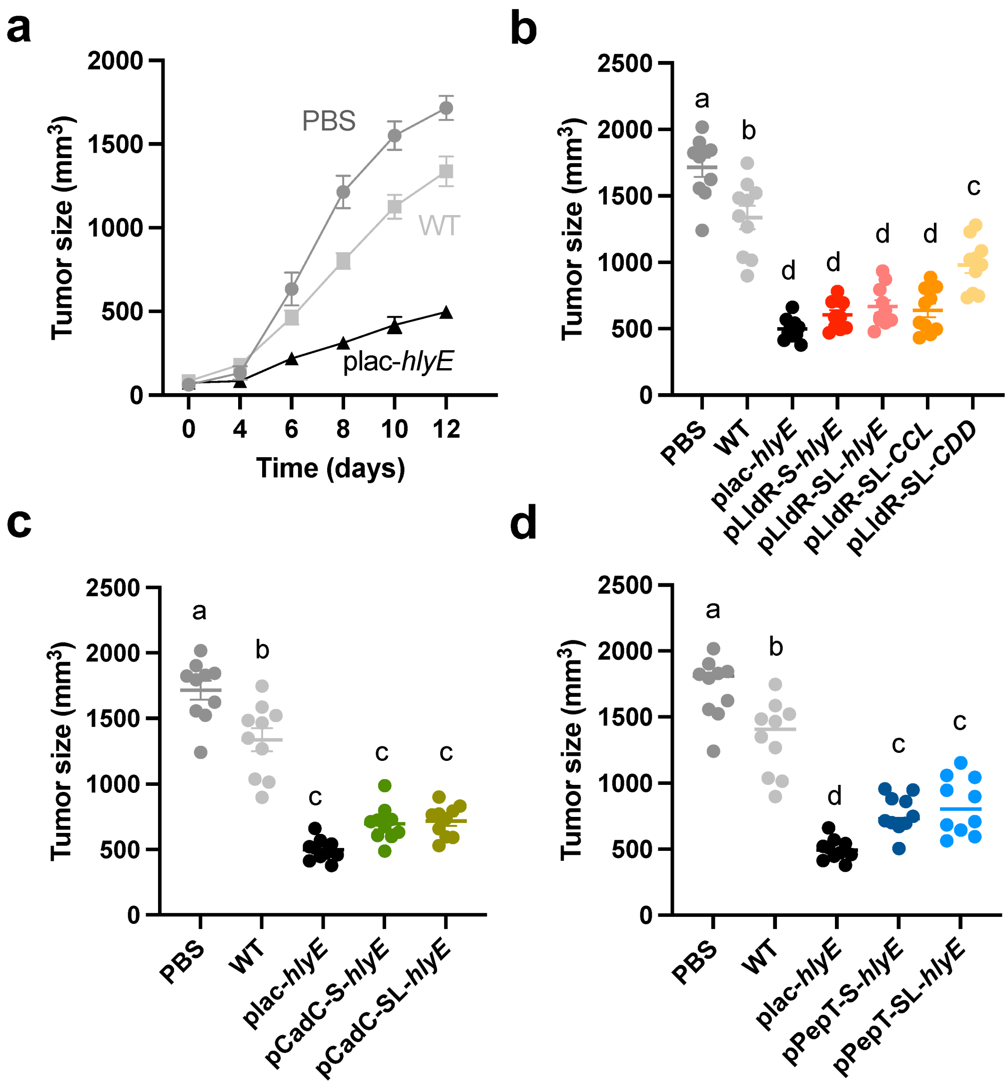

Supplemental Figure 9. Anti-tumor efficacy of the engineered EcNs in the CT26 homograft mouse model. a, Increased sizes of CT26-derived tumors were recorded from 150 mm^3. Mice (n = 10 in each group) were subcutaneously inoculated with 5 × 10^6 CT26 cells in the rear right flank and then received 20 μL 0.9% saline (control), 2 × 10^6 c.f.u/head constitutive therapeutic EcN (plac-*hlyE*) or wild type EcN. **b-d**, Comparison of tumor size extracted from each treatment group after mouse sacrifice (One-way ANOVA with Tukey post-test; significantly different groups are marked with different lowercase letters). The "S" in the legend represents the integration of the XOR Switch part of the engineered EcN, and the "L" represents the addition of the lysis gene (*φ*X174E).

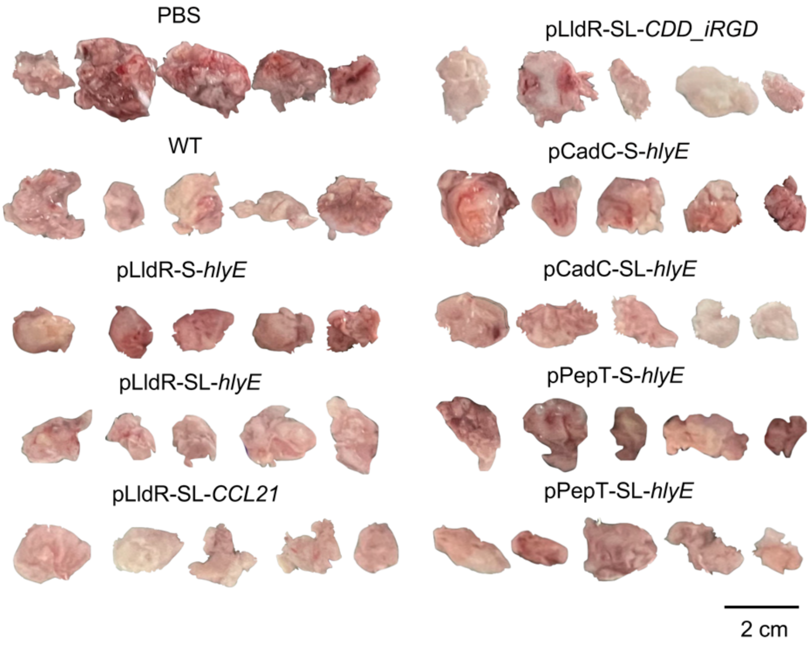

Supplemental Figure 10. Representative extracted tumor tissues from each treatment group. The "S" in the legend represents the integration of the XOR Switch part of the engineered EcN, and the "L" represents the addition of the lysis gene (φX174E). The scale bar labeled in the lower right corner of the image represents 2 cm.

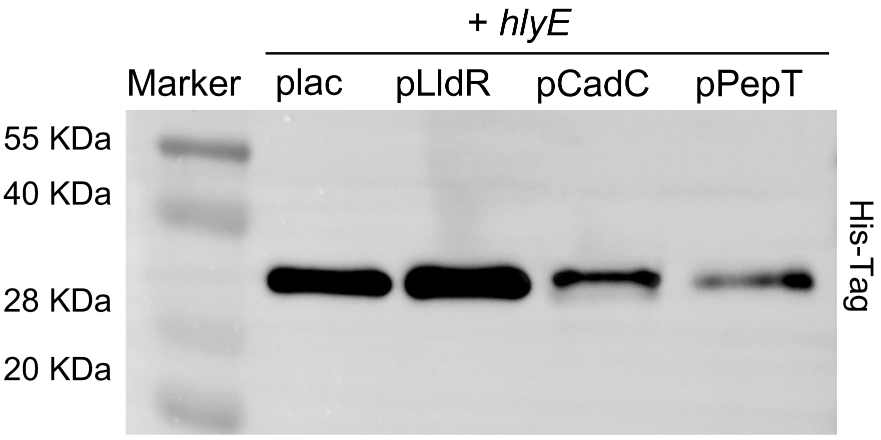

Supplemental Figure 11. Western blot showed the expression of *hlyE* in tumor tissues. Different therapeutic strains were administered to subcutaneous tumors of mice by intratumorally injection. Tumor samples were collected 12 hours later and analyzed for HlyE protein expression.

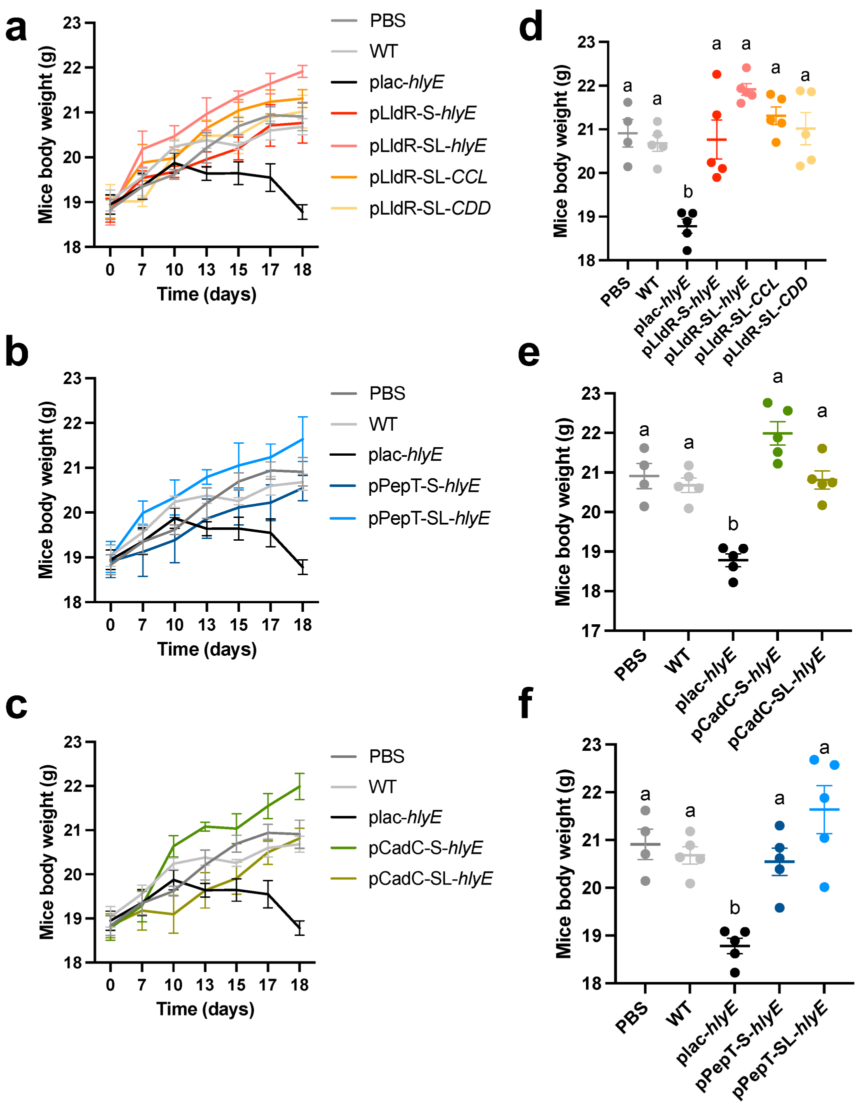

Supplemental Figure 12. Body weight analysis of subcutaneous tumor model mice. **a-c**, Mice body weight over time for subcutaneous tumor bearing mice with tumor injection of therapeutic strains (n = 5 mice). In the legend, "S" represents the engineering strain carrying XOR Switch gate, and "L" represents the engineering strain harboring lysis part. **d-f**, Comparison of mice body weight from each treatment group after 18 days of CT26 homograft (One-way ANOVA with Tukey post-test; significantly different groups were marked with different lowercase letters).

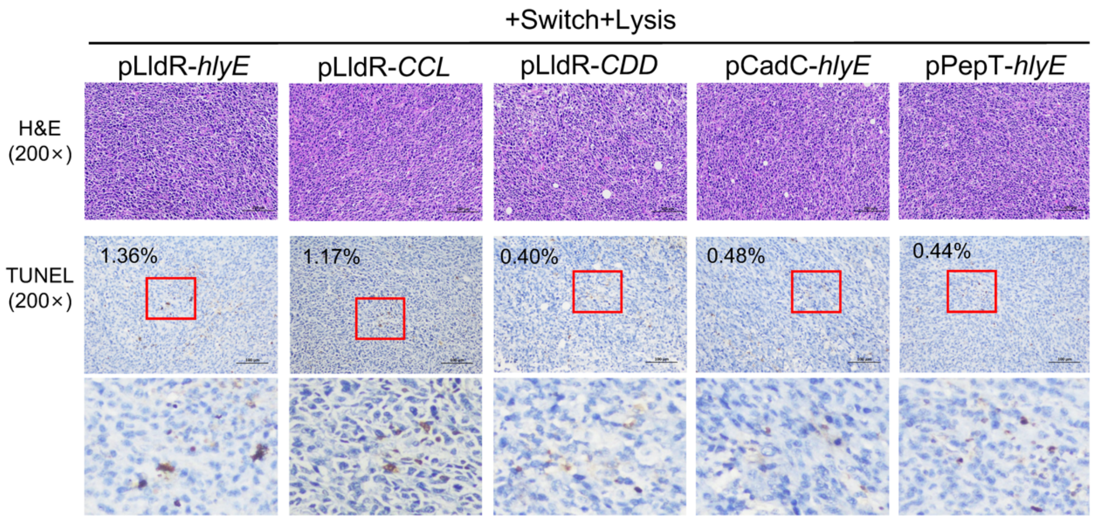

Supplemental Figure 13. H&E staining and TUNEL staining in the same tumor sections. Up: H&E staining images of tumor tissue from different treatment groups. Scale bar = 100 μm. Down: TUNEL staining images of tumor tissue from different treatment groups. Scale bar = 100 μm. The ratio of TUNEL-positive cells (brown cells) to total cells was analyzed using Aipathwell software and shown in the upper left of the images. The red box marks the magnified region, highlighting the representative area of positive cells in the TUNEL image and is displayed below.

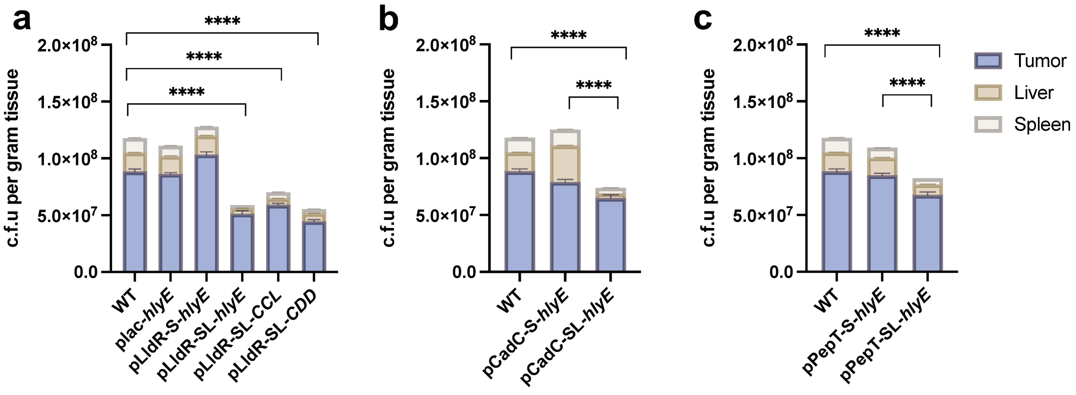

Supplemental Figure 14. Bacterial colonization of tissues in CT26 homograft model mice. Mice were euthanized at the end of the experiment. The tumor, liver and spleen were homogenized and plated on LB agar plates with antibiotics selection. Colonies were counted the next day. Absolute c.f.u. per gram of recovered bacteria from each gut compartment. n = 3 biological replicates. Data are presented as mean ±

s.e.m. Statistical analysis was performed for bacteria amount in tumor using One-way ANOVA with Tukey post-test. **** $p < 0.0001$.

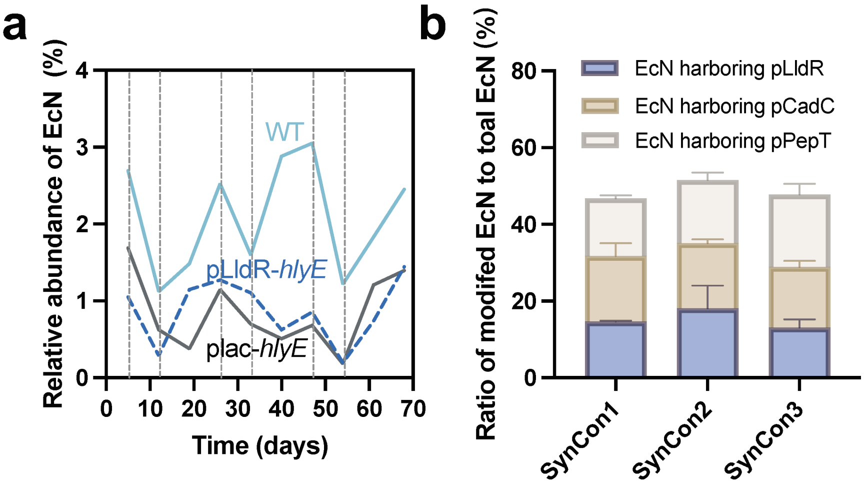

Supplemental Figure 15. Quantification analysis of EcNs in the intestinal of AOM/DSS model mice. a, Temporal dynamics of the EcNs population during whole experimental period (Black dashed lines represent the DSS treatment episodes). **b**, The ratio of SynCon members to total EcN were calculated using qPCR for fecal DNA analysis (n = 3, ± s.e.m).

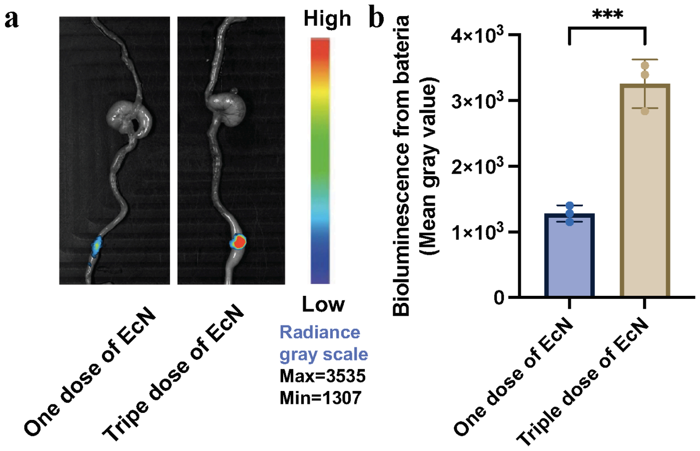

Supplemental Figure 16. *In vivo* bioluminescence analysis. a, Bioluminescence images of mice and their GI tract after 3 h post-oral gavage by EcN-lux (pSB1A3-plac-

luxCDABE). **b**, Comparison of bacterial bioluminescence intensity between two groups based on the average gray value (n = 3, ± s.e.m; Student's *t*-test; ***$p < 0.001$).

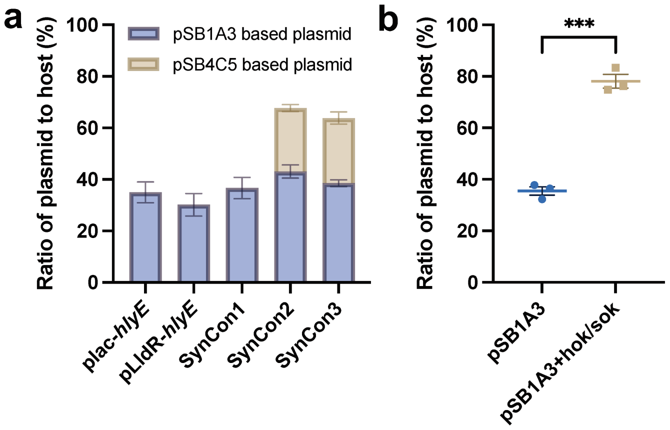

Supplemental Figure 17. Plasmid retention rates of single EcNs and SynCons in mouse gut. a, The proportion of plasmids to whole EcN were calculated using qPCR for fecal DNA analysis (n =3 biologically independent samples; data are presented as mean ± s.e.m). **b**, After 2 d of gavage, mice fecal DNA were analyzed using qPCR to assess the effect of hok/sok cassette on the plasmid loss rate of EcN carrying pSB1A3 in mouse intestine (n = 3, ± s.e.m; Student's *t*-test; ***$p < 0.001$).

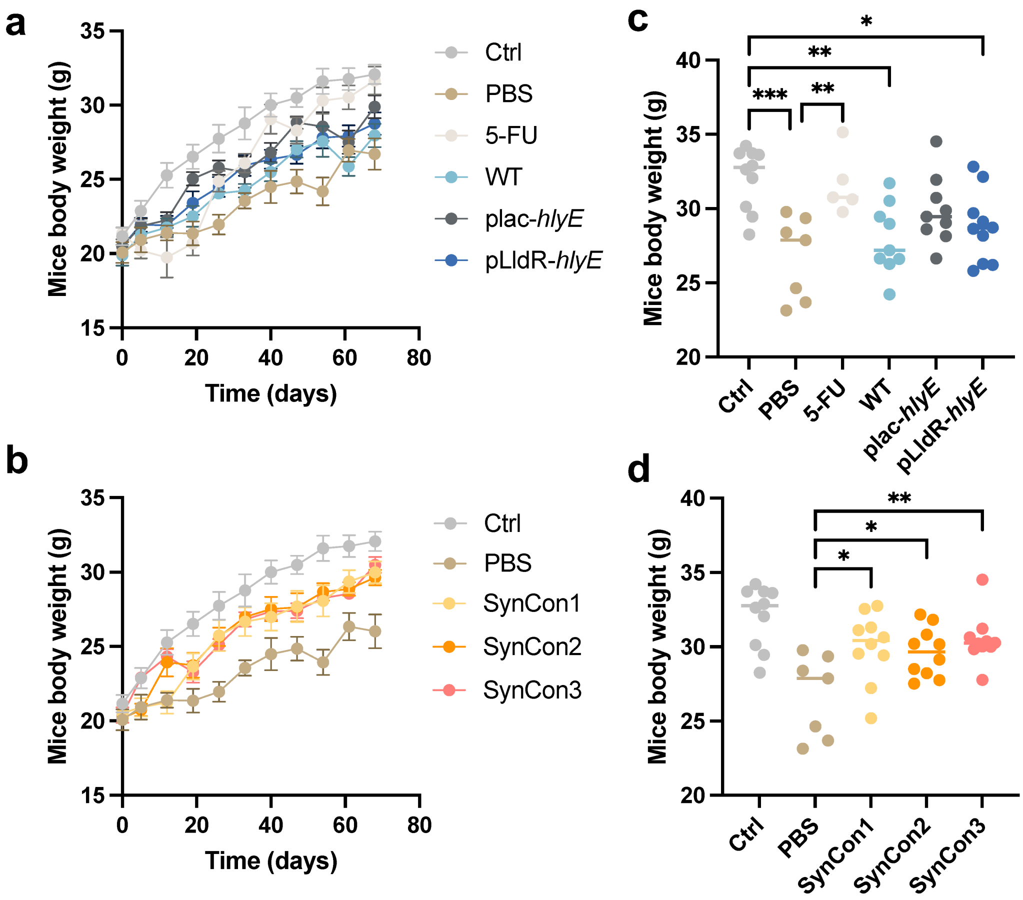

Supplemental Figure 18. Impact of different therapeutic regimens on the body weight of mice in the AOM/DSS mouse model. a-b, Changes in the bodyweight of mice were recorded every week. **c-d**, Comparison of mice body mice from each therapeutic group after 68 days AOM treatment (One-way ANOVA with Tukey post-test; *$p < 0.05$, **$p < 0.01$, ***$p < 0.001$).

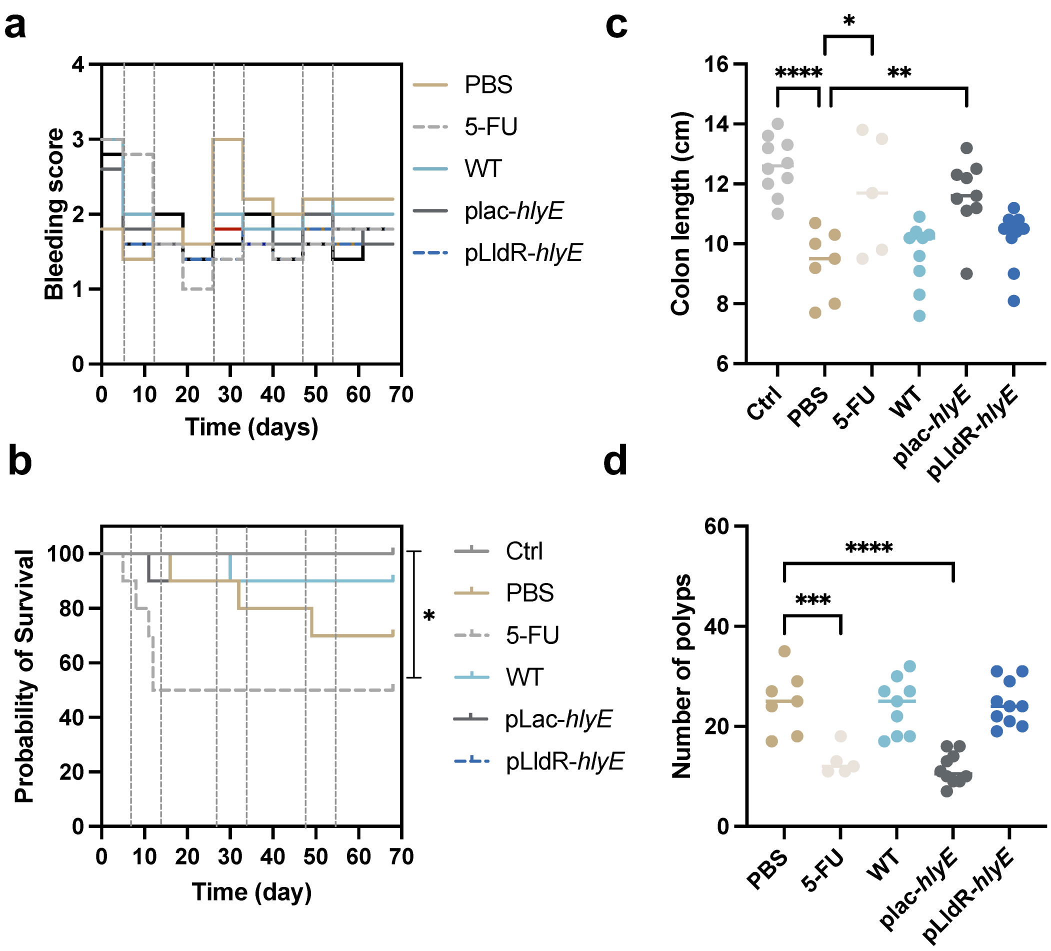

Supplemental Figure 19. Assessing the severity of colitis in AOM/DSS model mice subjected to different therapeutic regimens. a, Bleeding scores were assessed every week using hemoccult testing and visible signs. Dashed lines represent the DSS treatment episodes. **b**, Kaplan-Meier survival curves for mouse with different treatment groups. Log-rank test was performed to measure the statistical significance. $*p < 0.05$. Ctrl group did not receive AOM and DSS treatments. **c**, Colon length and **d** number of polyps were measured after 68 days (One-way ANOVA with Tukey post-test; $*p < 0.05$, $**p < 0.01$, $***p < 0.001$, $****p < 0.0001$).

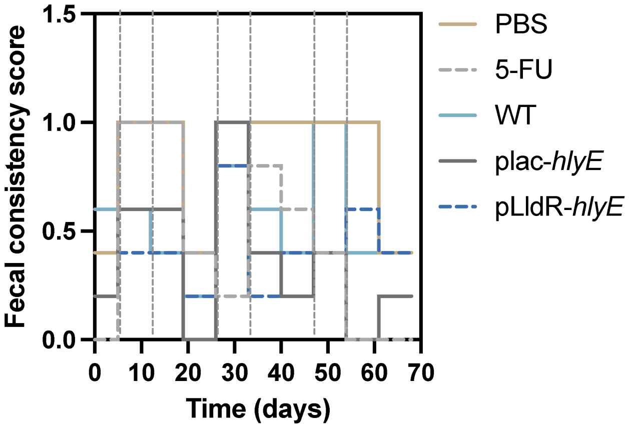

Supplemental Figure 20. Fecal consistency of AOM/DSS model mice treated with different therapeutic regimens.

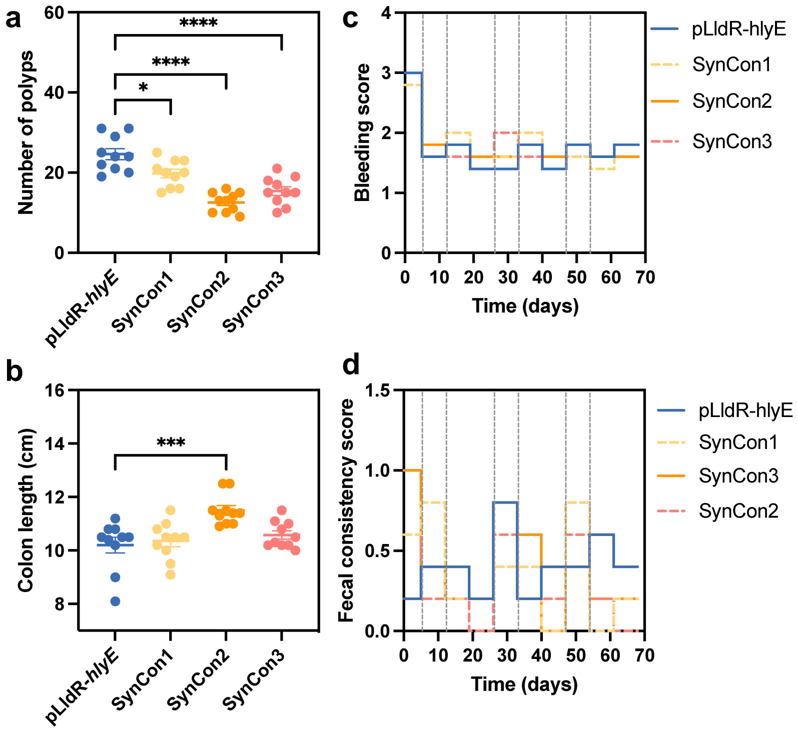

Supplemental Figure 21. The therapeutic effects of the single strain and SynCon strains on AOM/DSS model mice. Comparison of (**a**) the number of polyps (One-way ANOVA with Tukey post-test; *$p < 0.05$, ****$p < 0.0001$); (**b**) colon length (One-way ANOVA with Tukey post-test; ***$p < 0.001$); (**c**) occult blood score; and (**d**) fecal consistency score among pLldR-*hlyE* and SynCons groups.

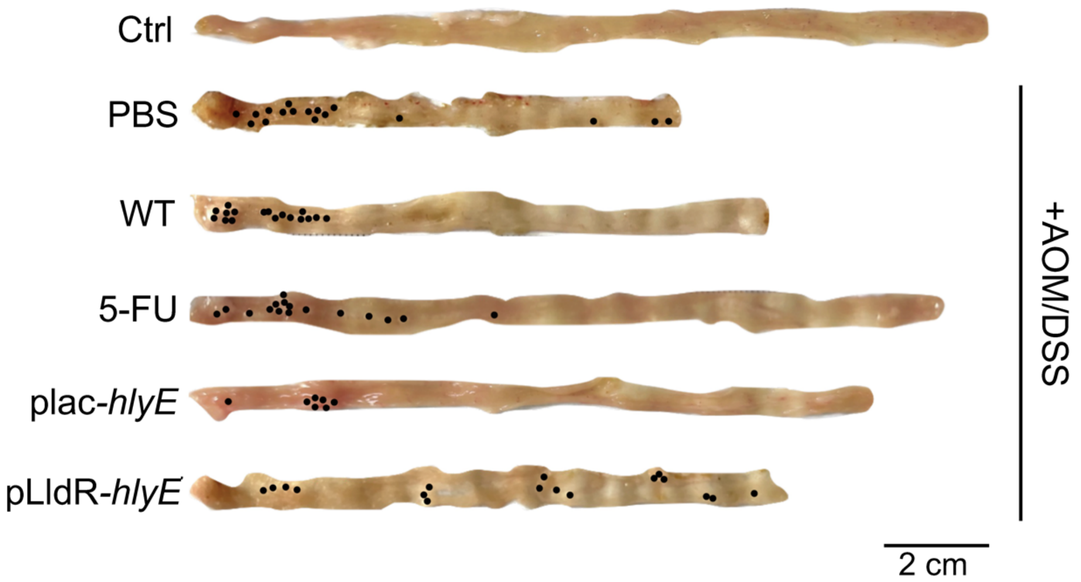

Supplemental Figure 22. Macroscopic appearance of colons in AOM/DSS model mice received different treatment. Black dots denote visible tumors. Scale bar (2 cm) labeled at bottom right of images.

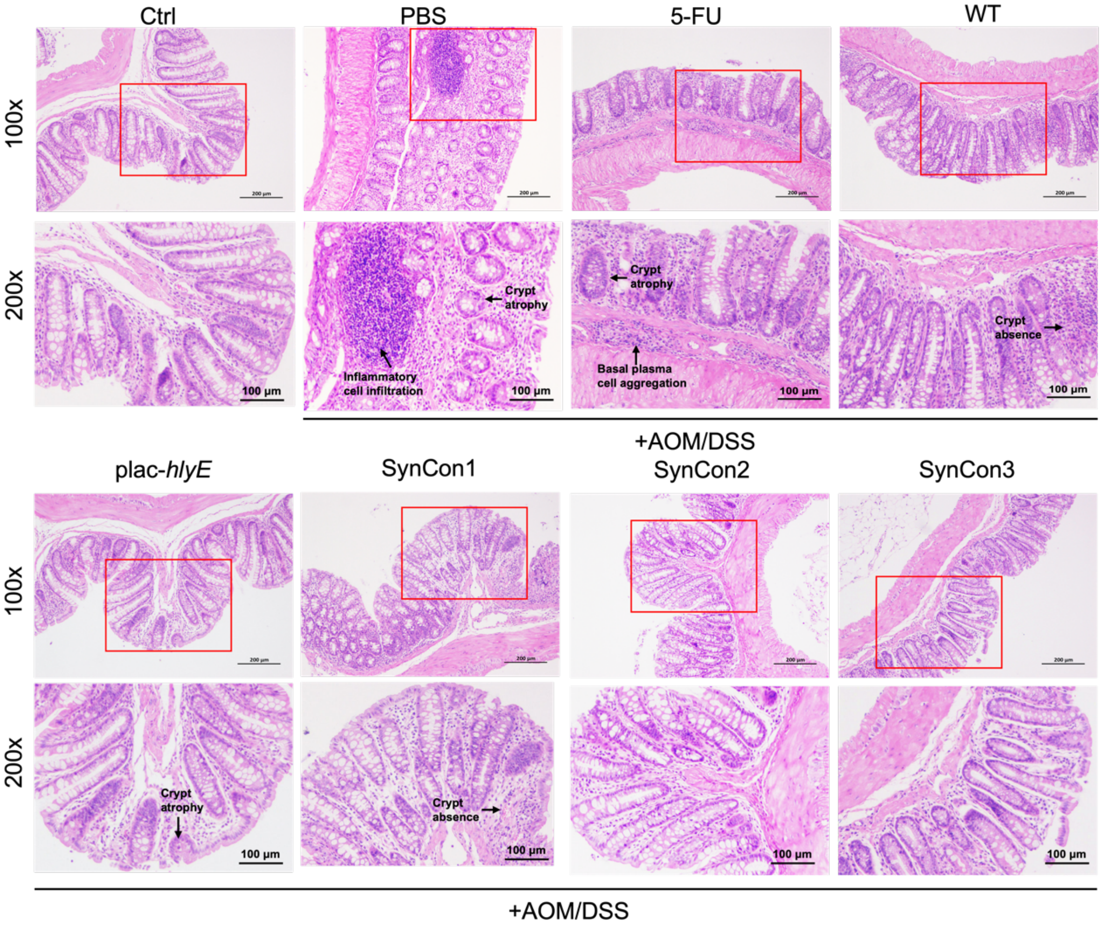

Supplemental Figure 23. H&E staining for colon sections taken from AOM/DSS model mice with different treatments. Each group provides 100x and 200x magnification of images, respectively. The red box indicates the enlarged portion. The scale is in the lower right corner of the image. Scale bar labeled in right corner represents 200 μm for 100x magnification and 100 μm for 200x magnification.

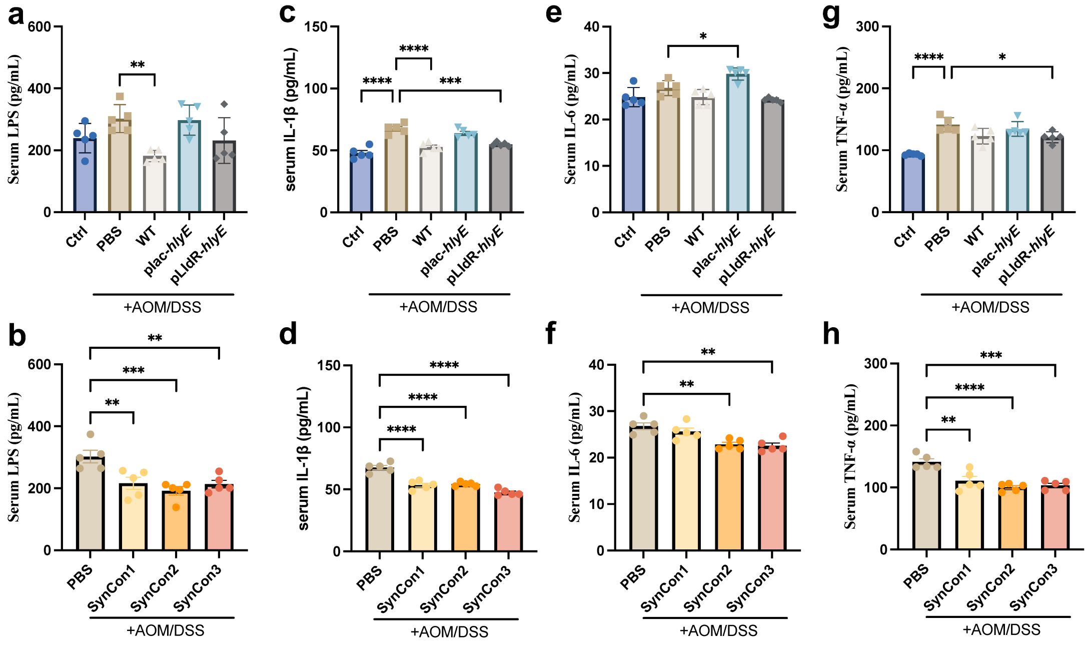

Supplemental Figure 24. Effects of dietary different EcNs and SynCons on the serum contents of the AOM/DSS-induced CRC mice. The levels of **(a-b)** Lipopolysaccharides (LPS), pro-inflammatory cytokines (**c-d**) IL-1β, (**e-f**) IL-6 and (**g-h**) TNF-α in the serum of each group mice on day 68 were determined using ELISA. Data are presented as mean values ± s.e.m (n =5 biologically independent samples). Statistical analysis was performed using One-way ANOVA with Tukey post-test (*$p < 0.05$, ** $p < 0.01$, *** $p < 0.001$, and **** $p < 0.0001$).

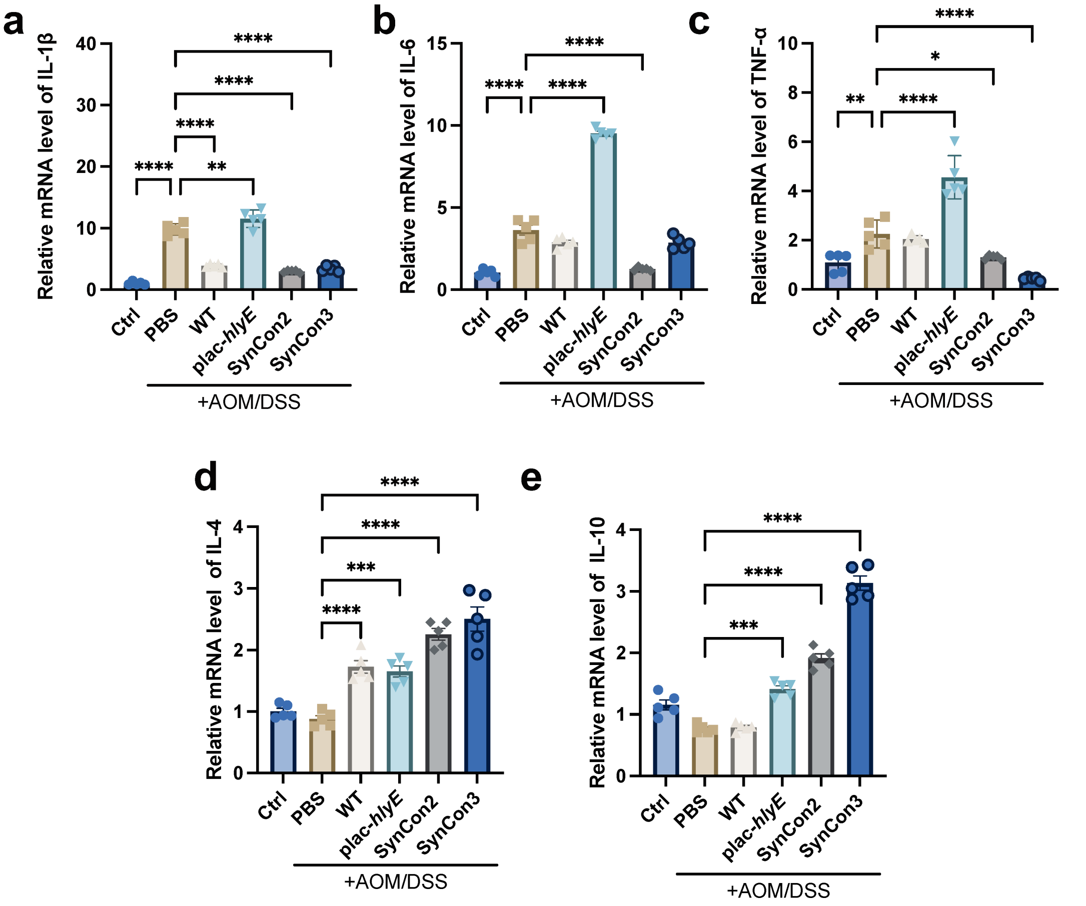

Supplemental Figure 25. Inflammation response of the mouse colon by treatment of AOM/DSS and administration of different EcNs and SynCons. The mRNA levels of pro-inflammatory cytokines (**a**) IL-1β, (**b**) IL-6, (**c**) TNF-α, or anti-inflammatory cytokines (**d**) IL-4, (**e**) IL-10 in mice colon were quantified using qRT-PCR (n = 5, ± s.e.m). Statistical analysis was performed using One-way ANOVA with Tukey post-test (*$p < 0.05$, ** $p < 0.01$, *** $p < 0.001$, and **** $p < 0.0001$).

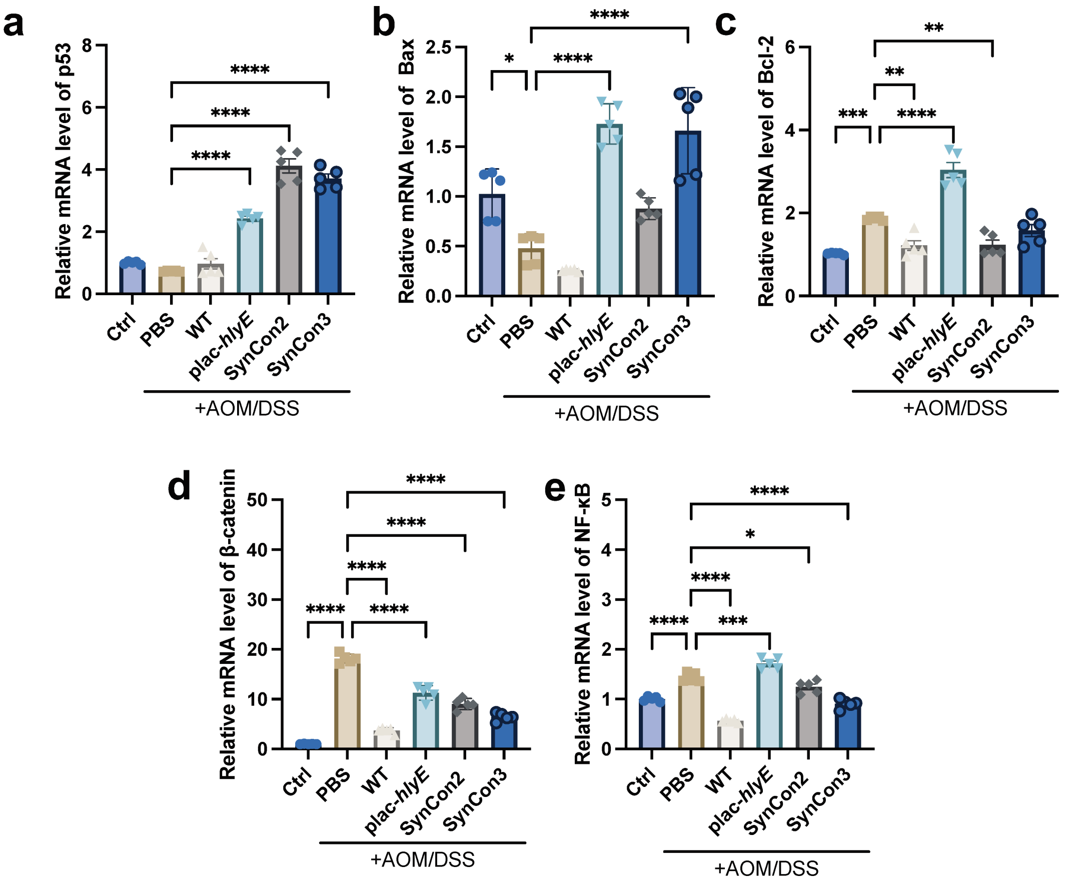

Supplemental Figure 26. Effects of dietary different EcNs and SynCons on colon tumorigenesis of the AOM/DSS-induced CRC mouse model. The mRNA levels of pro-apoptosis markers (**a**) p53, (**b**) Bax, anti-apoptosis markers (**c**) Bcl-2, and tumor cell proliferation markers (**d**) β-catenin, (**e**) NF-κB in mice colon were quantified using qRT-PCR (n = 5, ± s.e.m). Statistical analysis was performed using One-way ANOVA with Tukey post-test (*$p < 0.05$, ** $p < 0.01$, *** $p < 0.001$, and **** $p < 0.0001$).

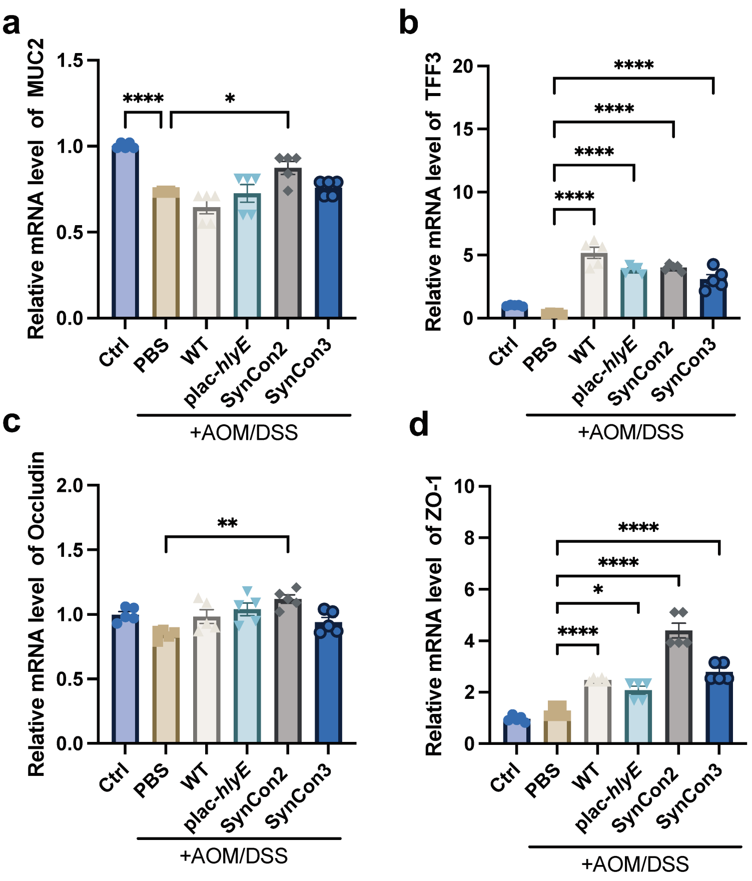

Supplemental Figure 27. Effects of different EcNs and SynCons on the gut barrier integrity of the AOM/DSS-induced CRC mouse model. The mRNA expression levels of (**a**) MUC2, (**b**) TFF3, (**c**) Occludin and (**d**) ZO-1 the mouse colon were measured using qRT-PCR (n = 5, ± s.e.m). Statistical analysis was performed using One-way ANOVA with Tukey post-test (**p* < 0.05, ** *p* < 0.01, and **** *p* < 0.0001).

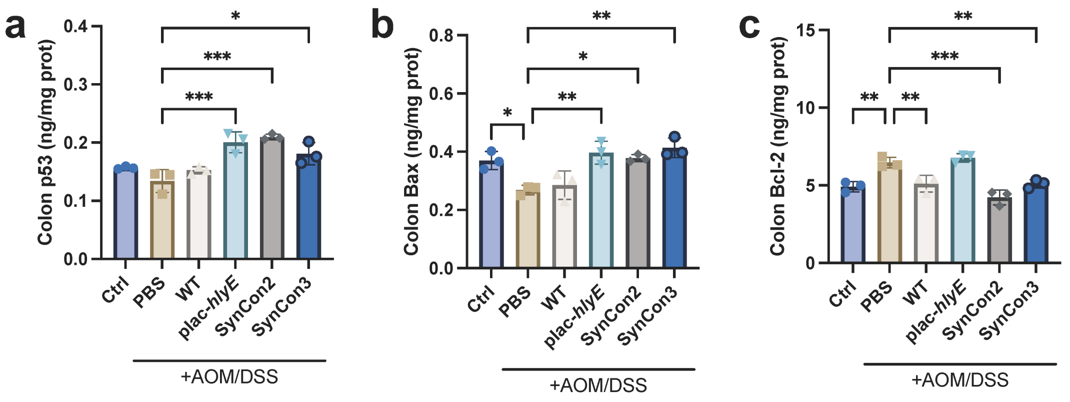

Supplemental Figure 28. Protein levels of tumor necrosis factor in distal colon samples across different groups. Expression levels of (**a**) p53, (**b**) Bax, and (**c**) Bcl-2

proteins in colon tissue. Apoptotic factor levels from ELISA were normalized using the total protein concentration determined by the Bradford assay kit (n = 3, ± s.e.m). Statistical analysis was performed using One-way ANOVA with Tukey post-test (*$p < 0.05$, ** $p < 0.01$, and *** $p < 0.001$).

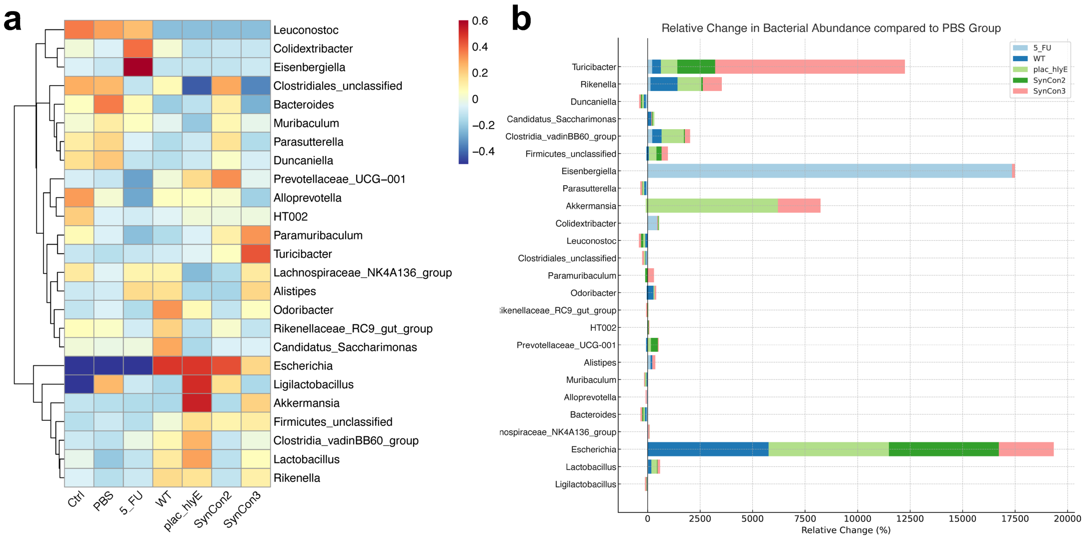

Supplemental Figure 29. Genus-level analysis of gut microbial composition. a, Heatmap exhibited relative abundance of gut microbial composition at top 25 genus level. **b**, Relative change in bacterial abundance compared to PBS group.

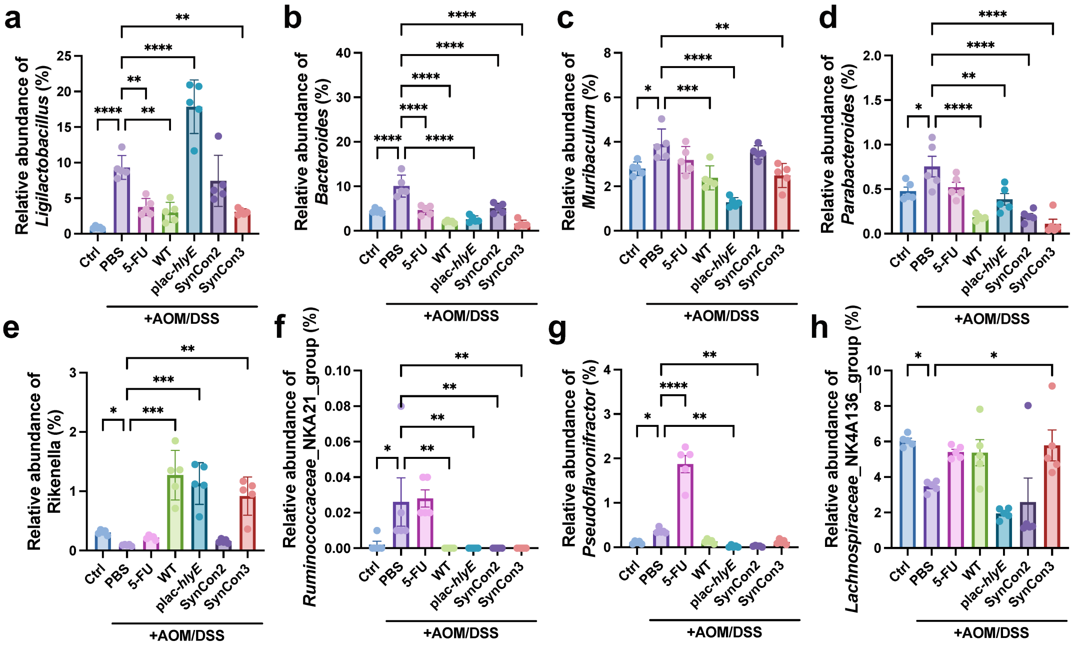

Supplemental Figure 30. Effects of different therapies on gut microbiota composition at genus level in AOM/DSS model mice. Comparison of relative abundance of (**a**) *Ligilactobacillus*, (**b**) *Bacteroides*, (**c**) *Muribaculum,* (**d**) *Parabacteroides*, (**e**) *Intestinimonas*, (**f**) *Ruminococcus*_NK4A214_group, (**g**) *Pseudoflavonifractor* and (**h**) *Lachnospiraceae*_NK4A136_group in the indicated groups (n = 5, ± s.e.m). Statistical analysis was performed using One-way ANOVA with Tukey post-test (*$p < 0.05$, ** $p < 0.01$, *** $p < 0.001$, and **** $p < 0.0001$).

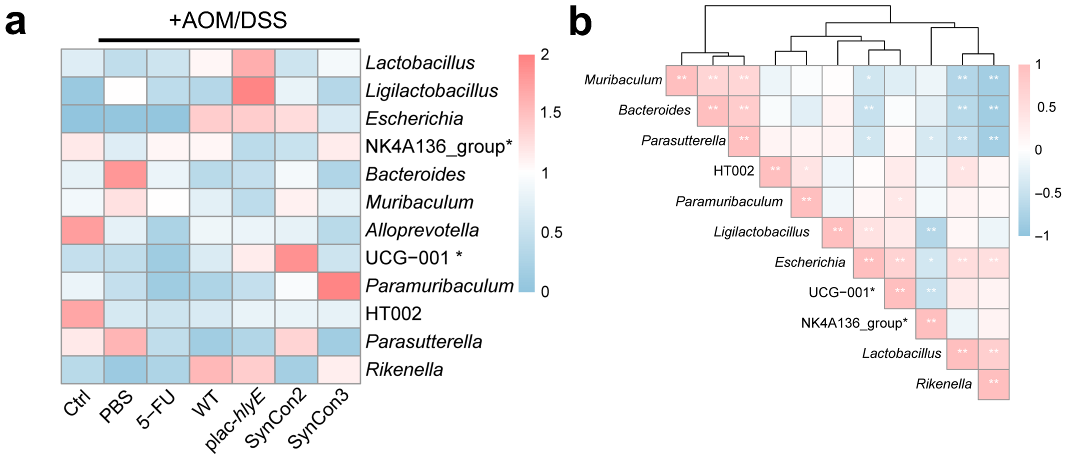

Supplemental Figure 31. The intestinal microbiota distribution at top 12 most abundant genus. a, The heatmap showed the difference of 12 most abundant genera in each group. NK4A136_group*: *Lachnospiraceae*_NK4A136_group; UCG-001*: *Prevotellaceae*_ UCG_001. **b**, Positive and negative correlation matrix between the top 12 most abundant genus taxa. Results of a pairwise Spearman's rank correlation. Correlations with adjusted p values less than 0.05 by the Benjamini-Hochberg FDR method are marked with white asterisk symbols. Related genera based on Euclidean distance were clustered together. Rubine, positive correlation; sapphire, negative correlation.

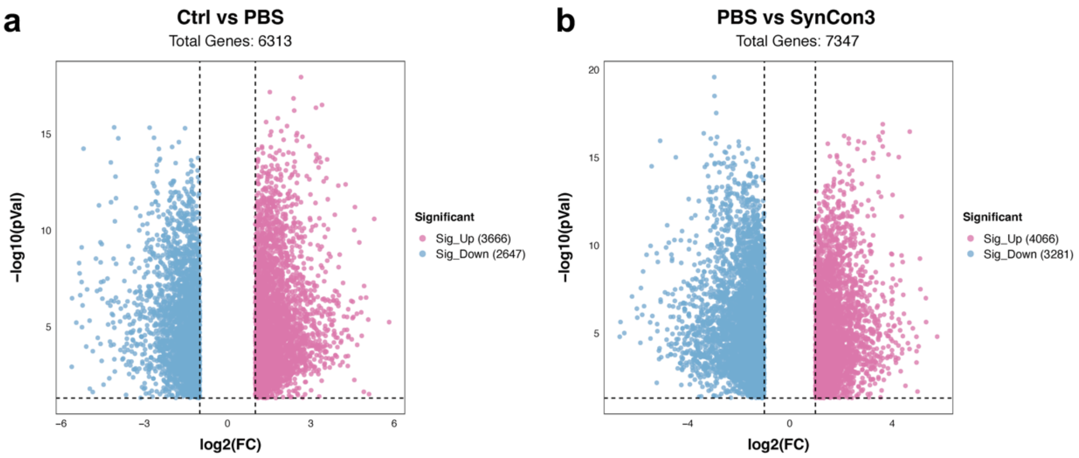

Supplemental Figure 32. Volcano plots of differential metabolites. a, The volcano plot displays differential metabolites between the Ctrl and PBS groups. **b**, The volcano plot illustrates differential metabolites between the PBS and SynCon3 groups. The x-axis represents the $\log_2$ transformation of the fold change (FC) of metabolite ions between the comparison groups, while the y-axis represents the -log10 transformation based on the Student's t-test *p*-value. Metabolites with an FC value greater than 2 and a *p*-value less than 0.05 are considered differential.

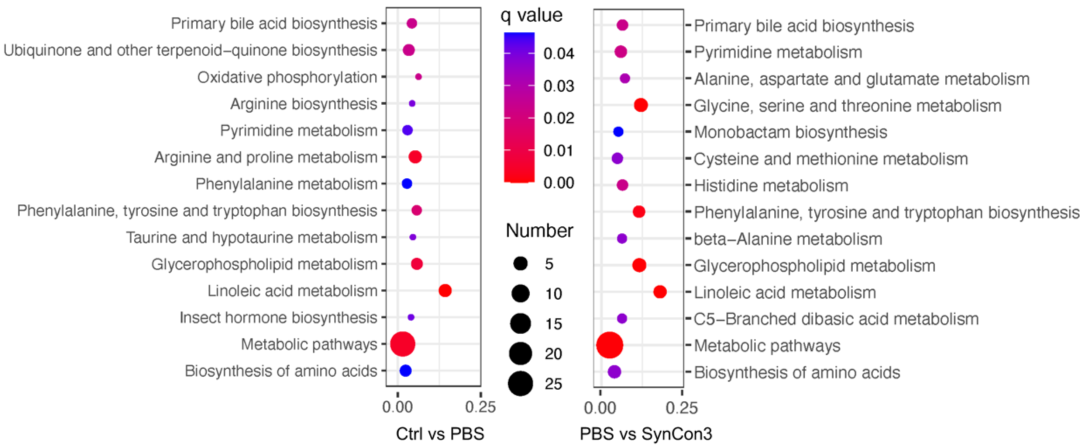

Supplemental Figure 33. KEGG pathway enrichment analysis of differential metabolites. The KEGG enrichment analysis was conducted using ggplot2, with results presented as a scatter plot. The q-value (adjusted *p*-value) is denoted by different colors; a smaller q-value indicates a higher degree of KEGG enrichment. 'Number' displays the count of enriched pathways. The x-axis represents the Rich Factor, indicating the relative abundance of differential metabolites in the pathway.

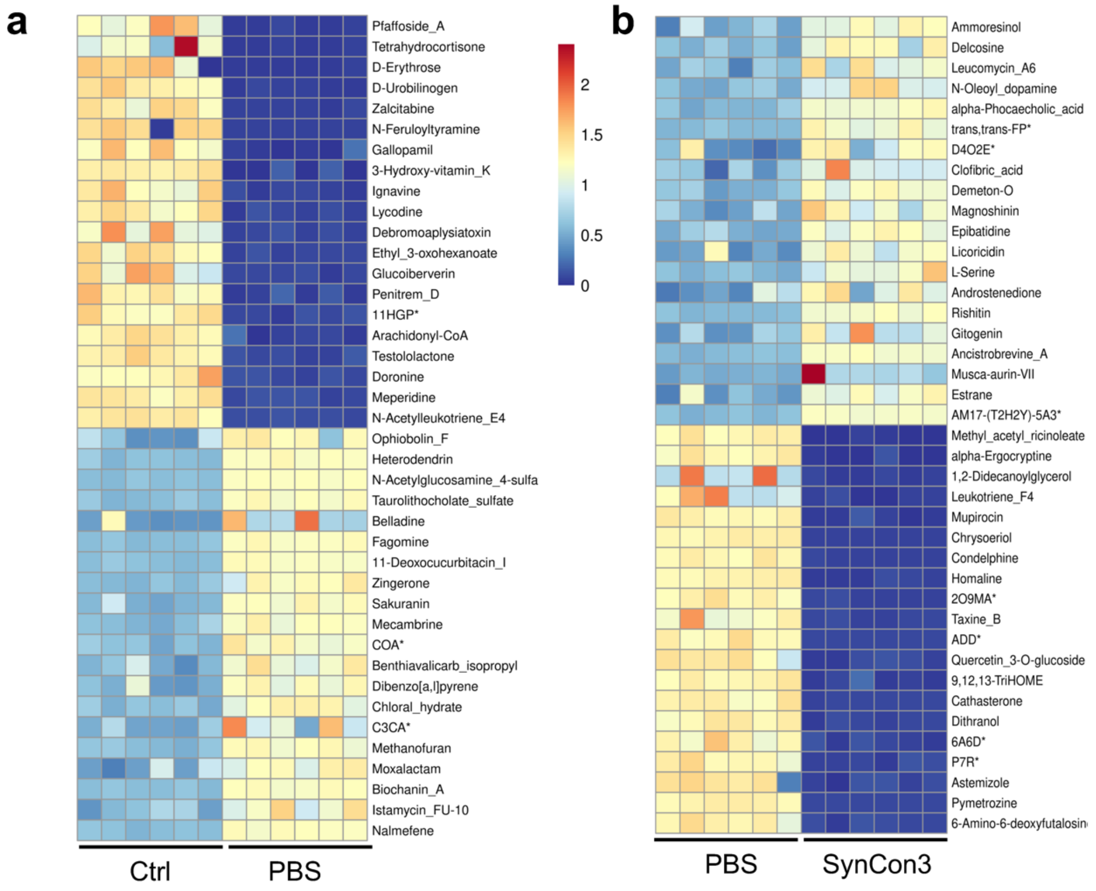

Supplemental Figure 34. Differential metabolites visualized in heatmaps. a, Heatmap illustrating the top 20 differential metabolites between the Ctrl and PBS groups. 11HGP*: 11-Hydroxyiridodial_glucoside_pentaacetate; COA*: (7R)-7-(5-Carboxy-5-oxopentanoyl) aminocephalosporinate; C3CA*: (3S,5S)-Carbapenam-3-carboxylic_acid. **b**, Heatmap showcasing the top 20 differential metabolites between the PBS and SynCon3 groups. trans,trans-FP*: trans,trans-Farnesyl_phosphate; D4O2E*: 2,5-Dichloro-4-oxohex-2-enedioate; AM17-(T2H2Y)-5A3*: 2alpha-Methyl-17beta-[(tetrahydro-2H-pyran-2-yl)oxy]-5alpha-androstan-3-one; 2O9MA*: 2-Oxo-9-methylthiononanoic_acid; ADD*: Androstane-3,17-diol_dipropionate; 6A6D*: 6-Amino-6-deoxyfutalosine; P7R*: Pinocembrin_7-rhamnosylglucoside. Differential metabolites are ranked based on fold change.

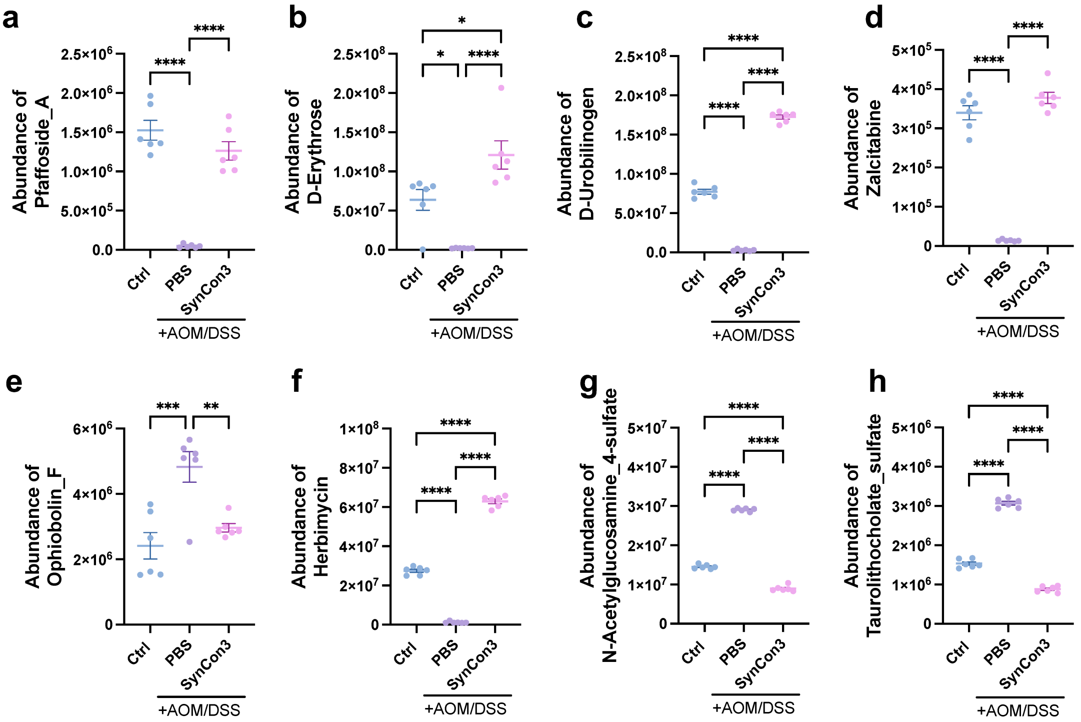

Supplemental Figure 35. Effect of AOM/DSS and dietary SynCon3 on fecal metabolome composition. Comparison of the abundance of (**a**) Pfaffoside A, (**b**) D-Erythrose, (**c**) D-Urobilinogen, (**d**) Zalcitabine, (**e**) Ophiobolin F, (**f**) Heterodendrin, (**g**) N-Acetylglucosamine_4-sulfate and (**h**) Taurolithocholate sulfate in the indicated groups (n = 6, ± s.e.m). Statistical analysis was performed using One-way ANOVA with Tukey post-test (*$p < 0.05$, ** $p < 0.01$, *** $p < 0.001$, and **** $p < 0.0001$).

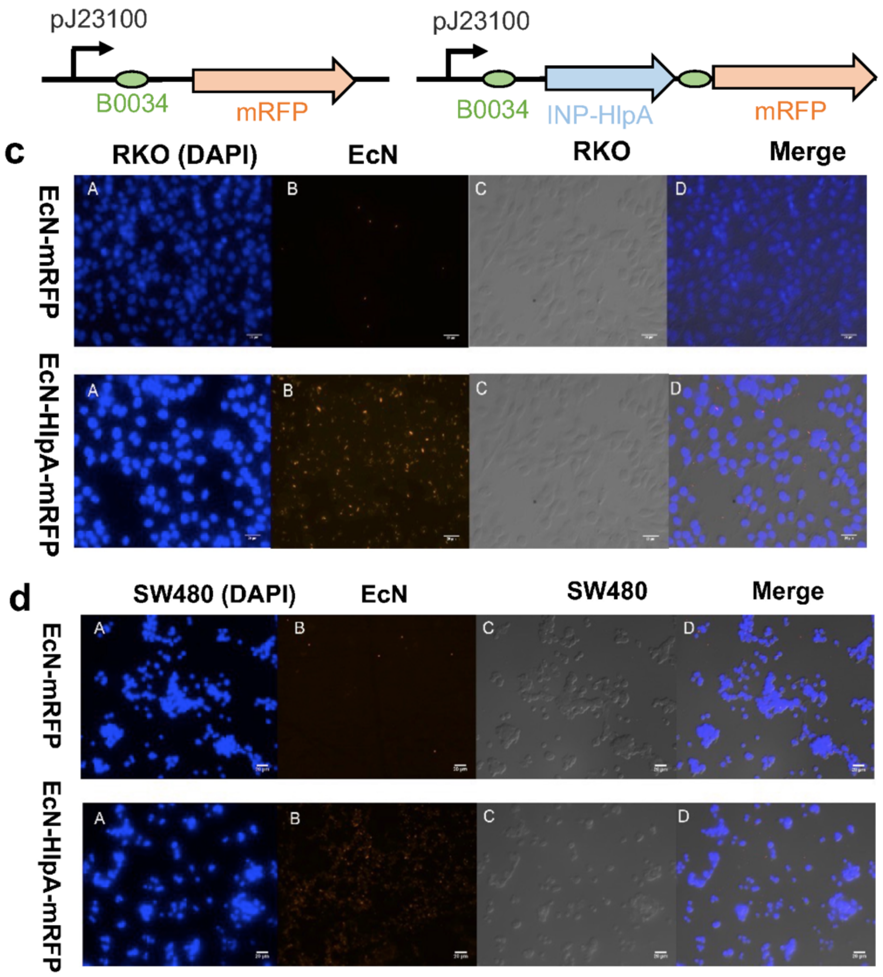

Supplemental Figure 36. HlpA enhanced the adhesion effect of EcN on tumor cells. **a-b**, Schematic diagram of control plasmid (constitutive expression of mRFP) and INP-HlpA integrate plasmid. **c-d**, The adhesion level of modified EcN to RKO cell and SW480 was observed by bacteria red fluorescence. Blue fluorescence represents DAPI-stained CRC cells.

III. Supplemental Tables

Supplemental Table 1. Linear discriminant analysis effect size between Ctrl and PBS groups after the last DSS administration.

Biomarker names	Groups	LDA values
Ligilactobacillus	PBS	4.64
Dubosiella	PBS	3.15
Intestinimonas	PBS	3.14
Roseburia	PBS	3.15
Pseudoflavonifractor	PBS	3.07
Bacteroides	PBS	4.48

*Ruminococcaceae*_NK4A214_group	PBS	3.05
Parabacteroides	PBS	3.16
Muribaculum	PBS	3.70
Catenibacterium	PBS	3.01
Parasutterella	PBS	3.25
Paraprevotella	Ctrl	3.40
HT002	Ctrl	3.90
Ruminococcus	Ctrl	3.39
Anaerotignum	Ctrl	3.02
Alloprevotella	Ctrl	4.24
Incertae_Sedis	Ctrl	3.23
*Lachnospiraceae*_NK4A136_group	Ctrl	4.05
Desulfovibrio	Ctrl	3.41
Rikenella	Ctrl	3.09
Bifidobacterium	Ctrl	3.17

Supplemental Table 2. Modified information of recombination plasmids.

Identifier	Promoter	Coding gene	Backbone	Ori	Resistance
Reporter 1/01	pLldR variant 1	*mRFP*	pSB1A3	ColE1	AmpR
Reporter 1/02	pLldR variant 2	*mRFP*	pSB1A3	ColE1	AmpR
Reporter 1/03	pLldR variant 3	*mRFP*	pSB1A3	ColE1	AmpR
Reporter 1/04	pLldR variant 4	*mRFP*	pSB1A3	ColE1	AmpR
Reporter 1/05	pLldR variant 5	*mRFP*	pSB1A3	ColE1	AmpR
Reporter 1/06	pLldR variant 6	*mRFP*	pSB1A3	ColE1	AmpR
Reporter 1/07	pLldR variant 7	*mRFP*	pSB1A3	ColE1	AmpR
Reporter 1/08	pLldR variant 8	*mRFP*	pSB1A3	ColE1	AmpR
Reporter 1/09	pLldR variant 9	*mRFP*	pSB1A3	ColE1	AmpR
Reporter 1/10	pLldR variant 10	*mRFP*	pSB1A3	ColE1	AmpR
Reporter 2	pCadC	*mRFP*	pSB1A3	ColE1	AmpR
Reporter 3	pPepT-mRFP	*mRFP*	pSB1A3	ColE1	AmpR
Reporter 4	plac	*luxCDABE*	pSB3T5	p15A	TetR
Controller 1	pLldR-	*TP901*	pSB1A3	ColE1	AmpR
Controller 2	pCadC-TP901	*TP901*	pSB1A3	ColE1	AmpR
Controller 3	pPepT-TP901	*TP901*	pSB1A3	ColE1	AmpR
Controller 4	pLldR	*TP901-φX174E*	pSB1A3	ColE1	AmpR
Controller 5	pCadC	*TP901-φX174E*	pSB1A3	ColE1	AmpR
Controller 6	pPepT	*TP901-φX174E*	pSB1A3	ColE1	AmpR
Switch 1	pP7-XOR gate	*mRFP*	pSB4C5	pSC101	CmR
Switch 2	pP7-XOR gate	*hlyE*	pSB4C5	pSC101	CmR
Switch 3	pP7-XOR gate	*CCL21*	pSB4C5	pSC101	CmR
Switch 4	pP7-XOR gate	*CDD_iRGD*	pSB4C5	pSC101	CmR
Test effector 1	pLldR	*hlyE*	pSB1A3	ColE1	AmpR

Test effector 2	pCadC	*hlyE*	pSB1A3	ColE1	AmpR
Test effector 3	pPepT	*hlyE*	pSB1A3	ColE1	AmpR
Test effector 4	plac	*hlyE*	pSB1A3	ColE1	AmpR

Supplemental Table 3. Bacterial and mammalian stains used in this study.

Type	Bacteria/Cell line	Harboring plasmid	Resistance
Control	*Escherichia coli* Nissle 1917 (EcN)	N/A	N/A
Biosensor Strains	EcN B1/01	Reporter 1/01	AmpR
	EcN B1/02	Reporter 1/02	AmpR
	EcN B1/03	Reporter 1/03	AmpR
	EcN B1/04	Reporter 1/04	AmpR
	EcN B1/05	Reporter 1/05	AmpR
	EcN B1/06	Reporter 1/06	AmpR
	EcN B1/07	Reporter 1/07	AmpR
	EcN B1/08	Reporter 1/08	AmpR
	EcN B1/09	Reporter 1/09	AmpR
	EcN B1/10	Reporter 1/10	AmpR
	EcN B2/01	Reporter 2	AmpR
	EcN B3/01	Reporter 3	AmpR
	EcN B1/11	Controller 1 + Switch 1	AmpR+CmR
	EcN B2/02	Controller 2 + Switch 1	AmpR+CmR
	EcN B3/02	Controller 3 + Switch 1	AmpR+CmR
	EcN B1/12	Controller 4 + Switch 1	AmpR+CmR
	EcN B2/03	Controller 5 + Switch 1	AmpR+CmR
	EcN B3/03	Controller 6 + Switch 1	AmpR+CmR
Therapeutic strains	EcN T4	Test effector 4	AmpR
	EcN T1/01	Test effector 1	AmpR
	EcN T2/01	Test effector 2	AmpR
	EcN T3/01	Test effector 3	AmpR
	EcN T1/02	Controller 1 + Switch 2	AmpR+CmR
	EcN T2/02	Controller 2 + Switch 2	AmpR+CmR
	EcN T3/02	Controller 3 + Switch 2	AmpR+CmR
	EcN T1/03	Controller 4 + Switch 2	AmpR+CmR
	EcN T2/03	Controller 4 + Switch 2	AmpR+CmR
	EcN T3/03	Controller 4 + Switch 2	AmpR+CmR
	EcN T1/04	Controller 4 + Switch 3	AmpR+CmR
	EcN T1/05	Controller 4 + Switch 4	AmpR+CmR
	Mouse colorectal carcinoma CT26	N/A	N/A
	Human colorectal carcinoma RKO	N/A	N/A
	Human colorectal carcinoma SW480	N/A	N/A

Note: EcN B1/01-12, EcN B2/01-03, and EcN B3/01-03 denote biosensor strains regulated by pLldR, pCadC, and pPepT, correspondingly. Similarly, EcN T1/01-05, EcN T2/01-03, and EcN T3/01-03 encompass therapeutic strains under the control of pLldR, pCadC, and pPepT, respectively.

Supplemental Table 4. Synthetic microbiome used in this study.

**Label**	**Bacteria member**	**Population ratio**
SynCon1	EcN T1/01; EcN T2/01; EcN T3/01	1:1:1
SynCon2	EcN T1/02; EcN T2/02; EcN T3/02	1:1:1
SynCon3	EcN T1/03; EcN T2/03; EcN T3/03	1:1:1

Supplemental Table 5. Primers for qPCR.

**Target**	**Primers**	**Sequence**	**Product size**
Total bacteria[13]	Tot.F	GCAGGCCTAACACATGCAAGTC	340 bp
	Tot.R	CTGCTGCCTCCCGTAGGAGT	
EcN[14]	Muta9	GCGAGGTAACCTCGAACATG	313 bp
	Muta10	CGGCGTATCGATAATTCACG	
pLldR	pLldR.qF	TCTTCGCTTATCTGACCTCTGG	162 bp
	pLldR. qR	AGTCTGTTGCTCATCTCCTTGT	
pCadC	pCadC.qF	GTAATCTTATCGCCAGTTTGGTCTGGTCA	89 bp
	pCadC.qR	AAAATGAAATTAGGAGAAGAG	
pPepT	pPepT.qF	GCAGGGGTAAAAGTGACC	81 bp
	pPepT.qR	CGAAAAGTGAGGGTGACTGC	
pSB1A3 ori	1A3.qF	GCTCACGCTGTAGGTATC	182 bp
	1A3.qR	CGCTCTGCTAATCCTGTTA	
pSB4C5 ori	4C5.qF	TACATCAGATTCCTACCTACG	139 bp
	4C5.qR	TGAGAACGAACCATTGAGAT	

Supplemental Table 6. Primers for qRT-PCR[15,16].

**Target gene**	**Forward primer (5'→3')**	**Revere primer (5'→3')**
GAPDH	GACGGCCGCATCTTCTTGT	CAGTGCCAGCCTCGTCCCGTACAA

p53	CCCCTGTCATCTTTTGTCCCT	AGCTGGCAGAATAGCTTATTGAG
Bax	AGACAGGGGCCTTTTTGCTAC	AATTCGCCGGAGACACTCG
Bcl-2	GCTACCGTCGTGACTTCGC	CCCCACCGAACTCAAAGAAGG
β-catenin	TCTCCTTGGCTGGCCTTTCTA	GTCACACAGCCCTGTCAAGA
NF-κB	AGCTGATGTGCATCGGCAAGTG	GTAGCTGCATGGAGACTCGAACAG
TNF-α	CTGAACTTCGGGGTGATCGG	GGCTTGTCACTCGAATTTTGAGA
IL-1β	CAACCAACAAGTGATATTCTCCATG	GATCCACACTCTCCAGCTGCA
IL-6	AAGTCGGAGGCTTAATTACACATGT	CCATTGCACAACTCTTTTCTCATTC
IL-4	GGTCTCAACCCCCAGCTAGT	GCCGATGATCTCTCTCAAGTGAT
IL-10	CTTACTGACTGGCATGAGGATCA	GCAGCTCTAGGAGCATGTGG
MUC2	ATGCCCACCTCCTCAAAGAC	GTAGTTTCCGTTGGAACAGTGAA
TFF3	TAATGCTGTTGGTGGTCCTG	CAGCCACGGTTGTTACACTG
Occludin	TCTGCTTCATCGCTTCCTTAG	GTCGGGTTCACTCCCATTA
ZO-1	AGGACACCAAAGCATGTGAG	GGCATTCCTGCTGGTTACA

IV. Supplementary References

1 Courbet, A., Endy, D., Renard, E., Molina, F. & Bonnet, J. Detection of pathological biomarkers in human clinical samples via amplifying genetic switches and logic gates. *Sci Transl Med* **7**, 289ra283, doi:10.1126/scitranslmed.aaa3601 (2015).

2 Goers, L. *et al.* Whole-cell Escherichia coli lactate biosensor for monitoring mammalian cell cultures during biopharmaceutical production. *Biotechnol Bioeng* **114**, 1290-1300, doi:10.1002/bit.26254 (2017).

3 Lee, Y. H., Kim, J. H., Bang, I. S. & Park, Y. K. The membrane-bound transcriptional regulator CadC is activated by proteolytic cleavage in response to acid stress. *J Bacteriol* **190**, 5120-5126, doi:10.1128/jb.00012-08 (2008).

4 Crack, J. C. *et al.* Signal perception by FNR: the role of the iron-sulfur cluster. *Biochem Soc Trans* **36**, 1144-1148, doi:10.1042/bst0361144 (2008).

5 Stricker, J. *et al.* A fast, robust and tunable synthetic gene oscillator. *Nature* **456**, 516-519, doi:10.1038/nature07389 (2008).

6 Chen, Y., Kim, J. K., Hirning, A. J., Josić, K. & Bennett, M. R. SYNTHETIC BIOLOGY. Emergent genetic oscillations in a synthetic microbial consortium. *Science* **349**, 986-989, doi:10.1126/science.aaa3794 (2015).

7 Bonyadi, M. R. & Michalewicz, Z. Particle Swarm Optimization for Single Objective Continuous Space Problems: A Review. *Evol Comput* **25**, 1-54, doi:10.1162/EVCO_r_00180 (2017).

8 Din, M. O. *et al.* Synchronized cycles of bacterial lysis for in vivo delivery. *Nature* **536**, 81-85, doi:10.1038/nature18930 (2016).

9 Chien, T. *et al.* Enhancing the tropism of bacteria via genetically programmed biosensors. *Nat Biomed Eng* **6**, 94-104, doi:10.1038/s41551-021-00772-3 (2022).

10 Lou, C., Stanton, B., Chen, Y. J., Munsky, B. & Voigt, C. A. Ribozyme-based insulator parts buffer synthetic circuits from genetic context. *Nat Biotechnol* **30**, 1137-1142, doi:10.1038/nbt.2401 (2012).

11 Yu, H. *et al.* A method for Absolute Protein Expression Quantity Measurement Employing Insulator RiboJ. *Engineering* **4**, 881-887, doi:https://doi.org/10.1016/j.eng.2018.09.012 (2018).

12 Mutalik, V. K. *et al.* Precise and reliable gene expression via standard transcription and translation initiation elements. *Nat Methods* **10**, 354-360, doi:10.1038/nmeth.2404 (2013).

13 Castillo, M. *et al.* Quantification of total bacteria, enterobacteria and lactobacilli populations in pig digesta by real-time PCR. *Vet Microbiol* **114**, 165-170, doi:10.1016/j.vetmic.2005.11.055 (2006).

14 Blum-Oehler, G. *et al.* Development of strain-specific PCR reactions for the detection of the probiotic Escherichia coli strain Nissle 1917 in fecal samples. *Research in Microbiology* **154**, 59-66, doi:https://doi.org/10.1016/S0923-2508(02)00007-4 (2003).

15 Oh, N. S., Lee, J. Y., Kim, Y. T., Kim, S. H. & Lee, J. H. Cancer-protective effect of a synbiotic combination between Lactobacillus gasseri 505 and a Cudrania tricuspidata leaf extract on colitis-associated colorectal cancer. *Gut Microbes* **12**, 1785803, doi:10.1080/19490976.2020.1785803 (2020).

16 Wang, T. *et al.* Lactobacillus coryniformis MXJ32 administration ameliorates azoxymethane/dextran sulfate sodium-induced colitis-associated colorectal cancer via reshaping intestinal microenvironment and alleviating inflammatory response. *Eur J Nutr* **61**, 85-99, doi:10.1007/s00394-021-02627-8 (2022).
